# DNA Repair Genes: Alternative Transcription and Gene Expression at the Exon Level in Response to the DNA Damaging Agent, Ionizing Radiation

**DOI:** 10.1371/journal.pone.0053358

**Published:** 2012-12-28

**Authors:** Helen B. Forrester, Jason Li, Daniel Hovan, Alesia N. Ivashkevich, Carl N. Sprung

**Affiliations:** 1 Centre for Innate Immunity and Infectious Disease, Monash Institute for Medical Research, Monash University, Clayton, Victoria, Australia; 2 Division of Research, Peter MacCallum Cancer Centre, Melbourne, Victoria, Australia; University of Massachusetts Medical School, United States of America

## Abstract

DNA repair is an essential cellular process required to maintain genomic stability. Every cell is subjected to thousands of DNA lesions daily under normal physiological conditions. Ionizing radiation (IR) is a major DNA damaging agent that can be produced by both natural and man-made sources. A common source of radiation exposure is through its use in medical diagnostics or treatments such as for cancer radiotherapy where relatively high doses are received by patients. To understand the detailed DNA repair gene transcription response to high dose IR, gene expression exon array studies have been performed and the response to radiation in two divergent cell types, lymphoblastoid cell lines and primary fibroblasts, has been examined. These exon arrays detect expression levels across the entire gene, and have the advantage of high sensitivity and the ability to identify alternative transcripts. We found a selection of DNA repair genes, including some not previously reported, that are modulated in response to radiation. Detailed dose and time course kinetics of DNA repair transcription was conducted and results have been validated utilizing PCR methods. Alternative transcription products in response to IR were identified in several DNA repair genes including *RRM2B* and *XPC* where alternative initiation sites were found. These investigations have advanced the knowledge about the transcriptional response of DNA repair.

## Introduction

Humans are exposed to IR from a number of sources. One of the most common sources of exposure is through medical procedures. For example, IR is commonly used for imaging and cancer treatment. Relatively large doses, on the order of tens of Gray (Gy), are received during radiotherapy that is provided as a curative or palliative treatment for a large proportion of cancer patients. Other sources of IR exposure include natural background radiation, accidental and occupational exposures. IR can cause direct or indirect damage to DNA which can lead to double-strand breaks (dsbs), single-strand breaks, base damage and DNA-DNA and DNA-protein cross-links. DNA dsbs are a critical lesion since they can result in senescence, death, or if improperly repaired, potentially tumorigenesis [1]. However, DNA dsbs occur relatively infrequently compared to other types of DNA damage. Organisms have evolved complex and sometimes redundant ways to repair a variety of DNA damage using a number of different pathways. Many proteins are involved in the pathways which include mismatch repair (MMR), base excision repair (BER), nucleotide excision repair (NER), non-homologous end joining (NHEJ) and homologous recombination (HR).

The importance of these DNA repair genes is evident from consequential disease associated with a particular DNA repair gene dysfunction. Commonly, DNA repair deficient individuals show sensitivity to DNA damaging agents and are susceptible to cancer [2], [3]. Gene knockout studies have shown some DNA repair genes are critical for an organism’s survival since they can result in embryonic lethality [4], [5]. Deficiencies of many genes in DNA repair pathways have been characterized and often result in clinical pathologies. Mutations in DNA dsb repair genes, including *LigIV*, and *DNAPKcs* of the NHEJ pathway have been identified in humans and result in radiosensitivity [6], [7]. Hereditary non-polyposis colorectal cancer (HNPCC) can be due to defects in genes that are required for mis-match DNA repair pathway, such as *MSH2*, *HLH1* or *MSH6* [8], [9]. A number of diseases are the result of a defect in NER. These diseases include xeroderma pigmentosum (XP) which is due to a defect in one of approximately eleven XP associated genes (including *XPA, ERCC3 (XPB)*, *XPC*, and *POLH* (*XPV*)). Skin cancer commonly occurs in these patients who are unable to properly repair UV induced DNA damage [10]. Trichothiodystrophy (mutations in *ERCC2* and *ERCC3*) and Cockayne syndrome (includes mutations in *ERCC6* and *ERCC8* genes) are other NER-associated diseases with similarities to XP but characterized by slightly different phenotypes [11], [12]. Fanconi anemia is another DNA repair deficiency disease. There are at least 13 DNA repair genes that are associated with this disease for which affected patients are especially sensitive to inter-strand DNA cross-linking [13]. There are approximately 153 genes that are directly involved with DNA repair [14], [15]. It should be noted that there are many other additional factors which are associated with these DNA repair proteins, or contribute to the proper regulation of DNA damage repair.

Most DNA repair occurs relatively rapidly following DNA damage. Sensors and transducer proteins organize the effector proteins for repair. Very rapid DNA repair responses often occur through post-translational protein modifications. For example, phosphorylation [16] by PI3 protein kinases such as ATM, ATR and DNA-PK is a well known regulatory mechanism. However, DNA repair gene transcription is also modulated in response to DNA damage [17], [18], [19], [20], [21], [22]. Transcription has many levels of regulation ranging from initiation to processing and transport of the mature message. High density gene expression arrays containing probes for every exon offer a means to conduct a detailed survey of the whole transcriptome at the exon level which can reveal alternative transcription following DNA damage.

DNA repair dysfunction commonly leads to disease including cancer, therefore, understanding the molecular mechanisms of DNA repair genes in response to DNA damaging agents such as IR is critical to develop innovative treatments. Therefore, we have comprehensively assessed the transcription of the known DNA repair genes [14] in response to IR exposure utilizing an exon array platform where each gene is extensively covered by probes, thus yielding highly robust data and the ability to assess alternative transcription.

## Materials and Methods

### Cell Culture

Derivation of lymphoblast and primary fibroblast cell lines has been previously described [19], [23]. Transcriptional response using exon arrays for twelve individuals were analysed [19], [23], [24] at 0 and 10 Gy 4 hr post-IR for both Epstein Barr virus (EBV) transformed lymphoblastoid cell lines (LCLs) and primary fibroblasts. Four samples were used from each group for other time points and doses and two samples of unirradiated cells for baseline normalization. LCLs were grown in RPMI media supplemented with 10% FBS and gentamicin and incubated in a 5 percent CO_2_ humidified 37°C incubator. All patients have given written informed consent and studies have been approved by the Peter MacCallum Cancer Centre and Monash University ethics committee.

### RNA Isolation

Cells (1×10^7^) were pelleted and resuspended in 3 ml PBS. Equal volume of Trizol (Invitrogen, Carlsbad, CA, USA) was added, mixed and incubated at room temperature for 15 minutes followed by the addition of 200 µl of chloroform. The sample was centrifuged, the aqueous layer was collected and mixed with and equal volume of 70 percent ethanol and added onto a RNeasy column (Qiagen, Venlo, The Netherlands). The RNA extraction was continued by using the RNAeasy method (with DNAse treatment) as per manufacturer’s recommendation except starting with the addition of Buffer RW1 to the sample. RNA concentration and integrity was determined by analysing on a bioanalyzer (Agilent, Santa Clara, CA, USA). RNA was determined to be high enough quality if a minimum RIN of 8.5 was obtained.

### Exon Arrays

GeneChip Human Exon 1.0 ST Array analysis was performed as per the ‘GeneChip Whole Transcript (WT) Sense Target labelling assay Manual’ (Affymetrix, Santa Clara, CA, USA). The rRNA from 1 µg of total RNA was reduced using a RiboMinus Human/Mouse Transcriptome Isolation Kit (Invitrogen, Carlsbad, CA, USA). Assessment of array quality was determined using Expression Console (Affymetrix.com). Gene expression was assessed using R, normalized using RMA and analysed using significance analysis of microarrays (SAM [25]). Probe selection regions (PSRs) are regions of primer sets designated for exons or potential exons in particular genes. Note that PSR probe sets have probe specific fluorescent and therefore show different relative levels of fluorescence. Array data and normalized expression have been deposited in the gene expression omnibus database: accession number GSE41840.

### Transcriptional Validation

Primers were designed to candidate exons or genes using ‘primer 3′ or Primer-Blast on-line software (NCBI). cDNA was made from 1 µg RNA using Super Script III First-Strand Synthesis System for RT-PCR (Invitrogen, Carlsbad, USA) as per manufactures recommendation. Initially the RNA, dNTPs and random hexamers were heated to 65°C for 5 minutes and then incubated at 25°C for 5 minutes with a subsequent incubation of 50°C for 1 hour and a 70°C incubation for 15 minutes with first strand buffer (Invitrogen, Carlsbad, USA), 0.1 M DTT, 0.5 mM dNTPs, 250 ng of random hexamers, 40 units of RNaseOUT and 200 units of SuperScript III RT. PCR amplification was carried out using 1.25 Units Go Taq polymerase (Promega, Wisconsin, USA), 200 nM primers, 5 ng cDNA, with a cycling protocol of 95°C: 2 min; (95°C: 15 sec; 60°C: 45 sec; 72°C: 30 sec) ×30; 72°C: 5 min. Products were run on a 2 percent agarose gel to determine amplification of the proper sized product. Real-time PCR was performed using these primers under the following conditions: Sybr Green Master Mix (Applied Biosystems, United Kingdom) with 200 nM of each primer was mixed with 5 ng of cDNA. The cycling steps were as follows. 95°C: 10 min; (95°C: 15 sec; 60°C: 60 sec) ×40, with a melting temperature ramp following amplification. A robotic system was used to load a 384 well plate with a subsequent run on the ABI 7900 quantitative real time PCR machine. All samples were run in triplicate. Primers used are shown in Table S1.

### 5′ RNA Ligase-mediated Rapid Amplification of cDNA Ends (5′-RLM-RACE)

5′-RLM-RACE was performed using FirstChoice RLM-RACE kit (Ambion, Austin, TX, USA) recommended by the manufacturer except the CIP digested RNA was purified using RNAeasy kit (Qiagen, Venlo, The Netherlands). The *RRM2B* and *XPC* transcripts were amplified using semi-nested PCR (as recommended by Ambion) with forward (inner and outer) primers to the adaptor (provided with the FirstChoice RLM-RACE kit) and reverse gene-specific primers (Table S1).

### Sequencing of PCR Amplicons

PCR amplicons were separated on 2 percent agarose, bands were stabbed using a pipet tip, placed in 100 µl of water, and 1 µl was re-amplified. The re-amplified PCR product was cleaned-up using a Qiagen PCR product spin column. Big Dye terminator sequencing was performed using PCR primers and the transcription start sites at the nucleotide level were determined by sequence comparison (NCBI BLAST).

## Results

### DNA Repair Genes Modulated at the Transcription Level

**Figure 1:**
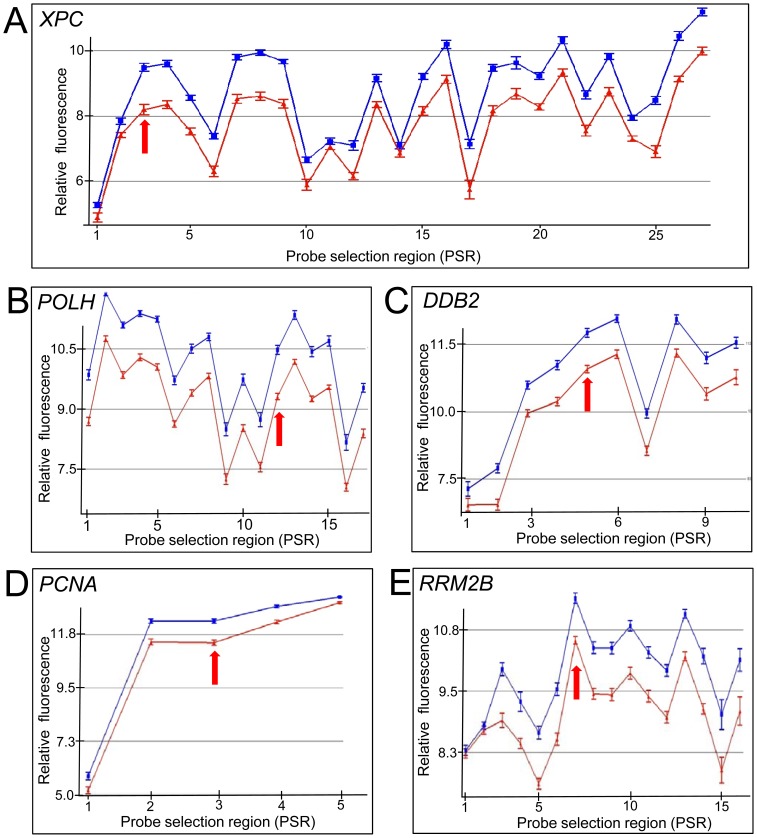
Induction of DNA repair genes at the exon level four hours after treatment with 10 Gy IR in LCLs. The top five DNA repair genes: *XPC* (A), *POLH* (B), *DDB2* (C), *PCNA* (D) and *RRM2B* (E) as identified using Partek Genomics Suite 6.6 statistical package. Relative fluorescence (y-axis; log_2_) is plotted for each PSR (x-axis). Core PSRs are labelled numerically in a 5′ to 3′ direction (left to right). Samples were either sham irradiated (red) or irradiated (blue) with 10 Gy from a ^137^Cs source. Arrow indicates the PSR region to which primers were designed for qRT-PCR used in figure 3A. Error bars = SEM (n = 12).

**Figure 2:**
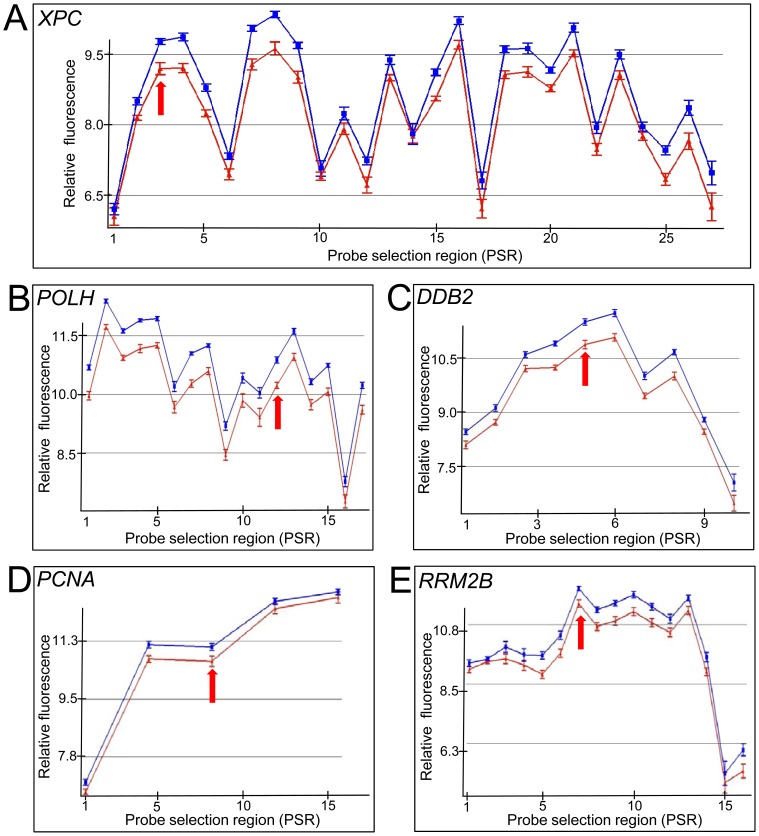
Induction of DNA repair genes at the exon level four hours after treatment with 10 Gy IR in primary fibroblast cells. The DNA repair genes: *XPC* (A), *POLH* (B), *DDB2* (C), *PCNA* (D) and *RRM2B* (E) as identified using Partek Genomics Suite 6.6 statistical package. Relative fluorescence (y-axis; log_2_) is plotted for each PSR (x-axis). Core PSRs are labelled numerically in a 5′ to 3′ direction (left to right). Samples were either sham irradiated (red) or irradiated (blue) with 10 Gy of radiation from a ^137^Cs source. RNA was collected 4 hours following treatment. Arrow indicates the PSR region to which primers were designed for qRT-PCR used in figure 3B. Error bars = SEM (n = 12).

**Figure 3:**
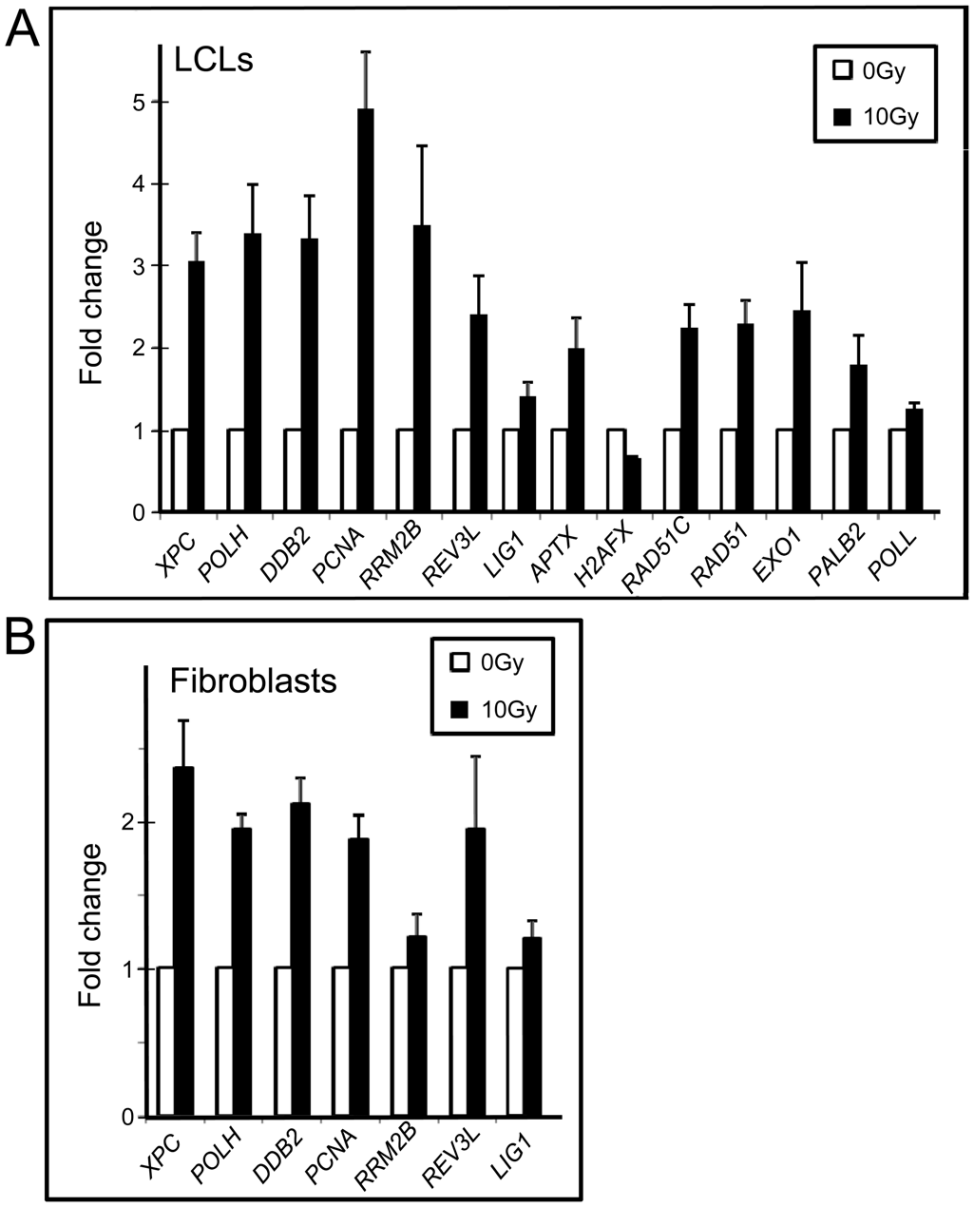
Validation of DNA repair gene expression modulation following 10 Gy IR using qRT-PCR. Ct values were normalized to *PGK*. Each bar represents data from 12 different cell lines for both LCL (A) and primary fibroblasts (B) with the following exceptions: 6 samples were used for *PCNA* and *RRM2B* in the LCL experiments; 10 samples were used for *XPC*, *RRM2B*, *REV3L* for the fibroblast experiments and 5 samples for, *EXO1*, *PALB2*, *LIG1* and *H2AFX* were used in the fibroblast experiments. Gene expression levels were averaged across multiple experiments. Four separate qRT-PCR runs were carried out for *POLH*, *DDB2*, *APTX*, *RAD51C*, and *PALB2* genes; three separate qRT-PCR runs were carried out for *PCNA*, *REV3L* and *EXO1* genes; and two separate qRT-PCR runs were carried out for *XPC*, *RRM2B*, *H2AFX*, and *RAD51* genes. Error bars = SEM (n = 12). Each value on an experiment was run in triplicate. All differences shown are statistically significant (p<0.05) using a t-test. PSRs used for amplification are: *XPC*: PSR853; *POLH*: PSR124; *DDB2*: PSR663; *PCNA*: PSR213; *RRM2B*: PSR293; *REV3L*: PSR729; *LIG1*: PSR905; *APTX*: PSR338; *H2AFX*: PSR185; *RAD51C*: PSR786; *RAD51*: PSR100; *EXO1*: PSR239; *PALB2*: PSR346; *POLL*: PSR904 for which some are indicated by and arrow in figures 1 and 2 and all full PSR numbers can be found in Figures S1 and S2.

The effect of IR on DNA repair gene expression in cells derived from two different lineages, LCLs and primary fibroblasts, was determined. Exon arrays were used to provide comprehensive probe coverage for all known DNA repair gene exons [14], [15]. The DNA repair genes that showed modulation were ranked based on ANOVA p-values (Tables 1 and 2). We found that 21 DNA repair genes were modulated in LCLs and 16 in primary fibroblasts, respectively when using a p-value cut-off of <0.05 comparing sham-irradiated to those irradiated with 10 Gy at 4 hrs post-IR. The top five genes (*XPC*, *POLH*, *DDB2*, *PCNA* and *RRM2B*) in lymphoblastoid cells, which were also induced in fibroblast cells, showed a clear gene expression induction at most exons across the gene (Figures 1 and 2, Profiles of all genes listed in the tables with probe selection regions noted are shown in Figures S1 and S2). These five genes have previously been identified as responsive to DNA damaging agents including IR [17], [19], [26], [27], [28], [29], [30], but these have not been well-characterized across the gene at the exon level which reveals underlying features of the transcripts with regard to different isoforms. Some DNA repair genes shown in Tables 1 and 2 (*LIG1, PALB2, CHAF1A*, and *MBD4)*, which have minor transcriptional changes at 4 hours post-IR, have not previously been recognized to be responsive to IR in this context. We validated the exon array expression data using qRT-PCR for many of the genes listed in Tables 1 and 2 with array data showing a 1.2 fold induction or greater in LCLs at 4 hr post-IR (Figure 3). qRT-PCR values were consistent with the exon array derived data, however, the amount of modulation was augmented when analysed with qRT-PCR. qRT-PCR was also used to determine the response to IR for other DNA repair genes that had p-values derived from the exon array data of >0.05 in LCLs. For example, *POLL* was shown to have a statistically significant induction (p<0.05) in response to IR in LCLs (Figure 3).

**Table 1:** DNA repair genes transcriptionally modulated in LCLs at 4 hr post-IR (p<0.05).

Gene	Rank	Total gene rank	Fold change	p-value	DNA repair pathway
*XPC*	1	17	2	1.4E−10	Nucleotide excision repair
*POLH*	2	42	2.2	8.2E−09	DNA polymerases (catalytic subunits)
*DDB2*	3	48	1.7	2.3E−08	Nucleotide excision repair
*PCNA*	4	54	1.5	4.6E−08	DNA polymerases (catalytic subunits)
*RRM2B*	5	69	1.8	2.1E−07	Modulation of nucleotide pools
*REV3L*	6	204	1.3	0.0001	DNA polymerases (catalytic subunits)
*LIG1*	7	298	1.2	0.0005	Nucleotide excision repair
*APTX*	8	383	1.2	0.001	Editing and processing nucleases
*TDP1*	9	411	−1.2	0.002	Repair of DNA-protein crosslinks
*H2AFX*	10	512	−1.2	0.003	Chromatin Structure
*CHAF1A*	11	538	1.1	0.004	Chromatin Structure
*MBD4*	12	638	−1.2	0.006	Base excision repair
*ATM*	13	672	−1.2	0.007	Genes defective in diseases associated with sensitivity to DNA damaging agents
*DCLRE1C*	14	691	−1.2	0.007	Non-homologous end-joining
*RAD51C*	15	696	1.4	0.007	Homologous recombination
*RAD51*	16	729	1.2	0.009	Homologous recombination
*EXO1*	17	850	1.2	0.013	Editing and processing nucleases
*RAD51L3*	18	946	1.1	0.018	Homologous recombination
*PALB2*	19	963	1.1	0.018	Genes defective in diseases associated with sensitivity to DNA damaging agents
*MDC1*	20	1179	−1.1	0.030	Other conserved DNA damage response genes
*RECQL4*	21	1403	1.1	0.042	Genes defective in diseases associated with sensitivity to DNA damaging agents

**Table 2:** DNA repair genes transcriptionally modulated in primary fibroblasts at 4 hr post-IR (p<0.05).

Gene	Rank	Total gene rank	Fold change	p-value	DNA repair pathway
*POLH*	1	12	1.6	4.1E−07	DNA polymerases (catalytic subunits)
*DDB2*	2	15	1.5	9.7E−07	Nucleotide excision repair
*XPC*	3	19	1.4	1.3E−06	Nucleotide excision repair
*RRM2B*	4	172	1.4	0.003	Modulation of nucleotide pools
*DCLRE1C*	5	272	−1.2	0.008	Non-homologous end-joining
*TDP1*	6	375	−1.2	0.014	DNA protein crosslinks
*DCLRE1A*	7	449	−1.2	0.018	DNA cross-link repair
*CHEK2*	8	485	−1.1	0.020	Effector kinase
*ALKBH2*	9	509	−1.1	0.022	Resistance to alkylation damage
*PCNA*	10	570	1.3	0.026	DNA polymerases (catalytic subunits)
*DUT*	11	592	−1.1	0.027	dUTPase
*UBE2A*	12	739	−1.2	0.035	Editing & processing nuclease
*XAB2*	13	751	1.1	0.036	Nucleotide excision repair
*MNAT1*	14	888	−1.1	0.044	Nucleotide excision repair
*NEIL3*	15	997	−1.4	0.049	Base excision repair
*APEX1*	16	998	−1.1	0.049	Base excision repair

### DNA Repair Gene Expression in Two Divergent Cell Types

**Figure 4:**
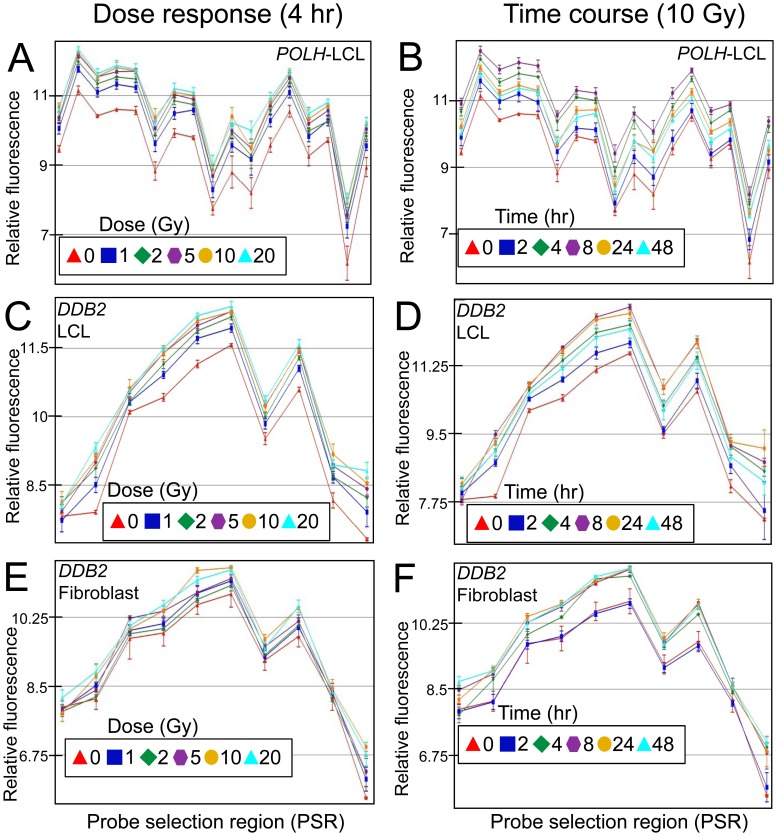
Dose response and time course of selected DNA repair genes in human cell lines. PSR hybridization signals are shown for a two DNA repair genes that are induced following radiation. These are *POLH* (A, B) and *DDB2* (C–F) in LCLs (A–D) or fibroblasts (E, F). Relative fluorescence is plotted on the y-axis and PSRs are plotted evenly across the x-axis in a 5′ to 3′ direction (left to right). Samples from different individuals were either sham irradiated (red) or irradiated with 1 Gy (blue), 2 Gy (green), 5 Gy (purple), 10 Gy (orange) or 20 Gy (aqua) of radiation (A, C and E; n = 4). RNA was collect 4 hours following treatment. Time course of DNA repair genes were either sham irradiated (red) or irradiated with 10 Gy and RNA isolated 2 hrs (blue), 4 hrs (green), 8 hrs (purple), 24 hours (orange) or 48 hours (aqua) after irradiation (B, D and F; n = 4). Error bars = SEM.

**Figure 5:**
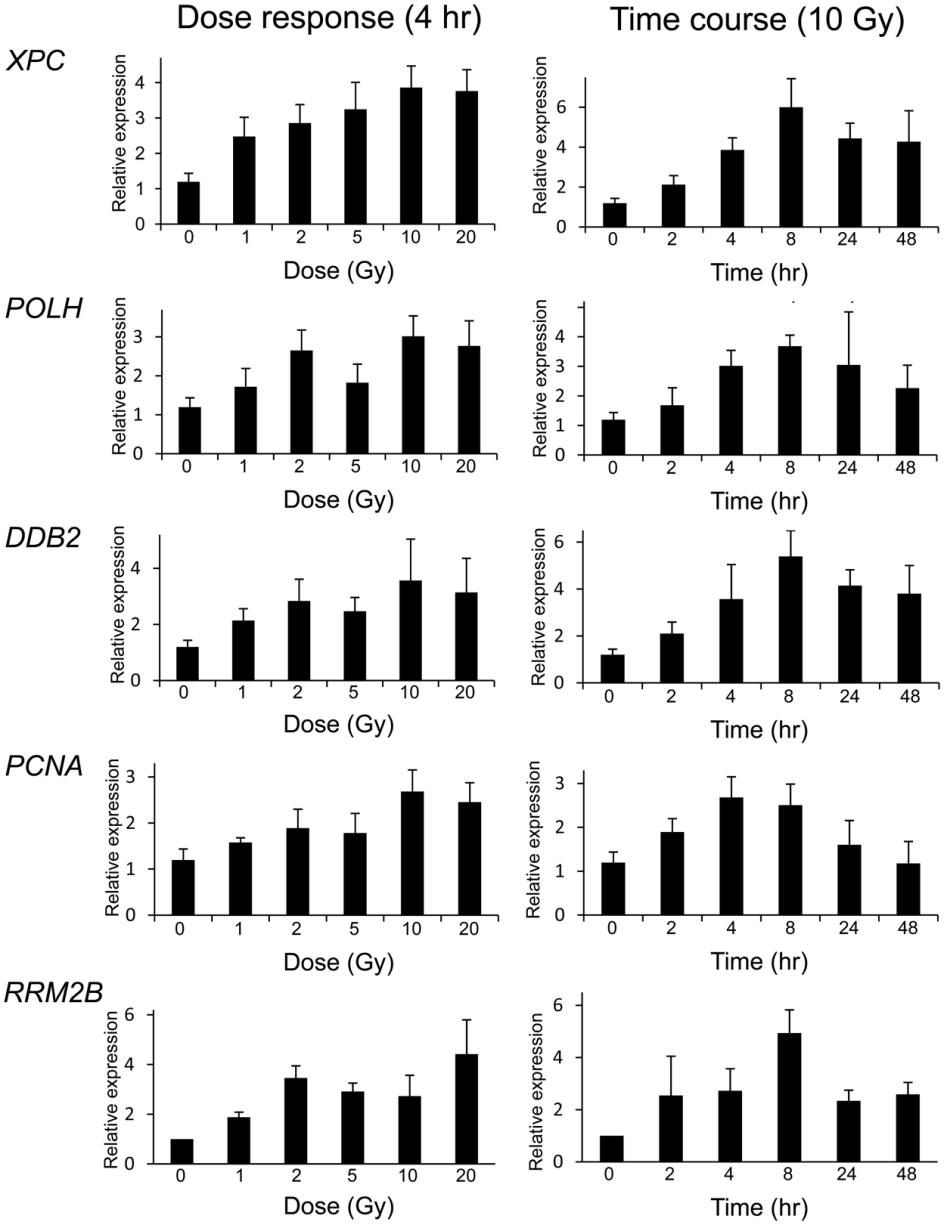
Expression levels of the top five DNA repair genes as determined by qRT-PCR in LCLs over different IR doses and times. Relative gene expression is shown for samples that were either sham irradiated or irradiated with 1 Gy, 2 Gy, 5 Gy, 10 Gy or 20 Gy of radiation at 4 hours post-IR, or sham irradiated or irradiated with 10 Gy at 2 h, 4 h, 8 h, 24 h or 48 h post-IR.

**Figure 6:**
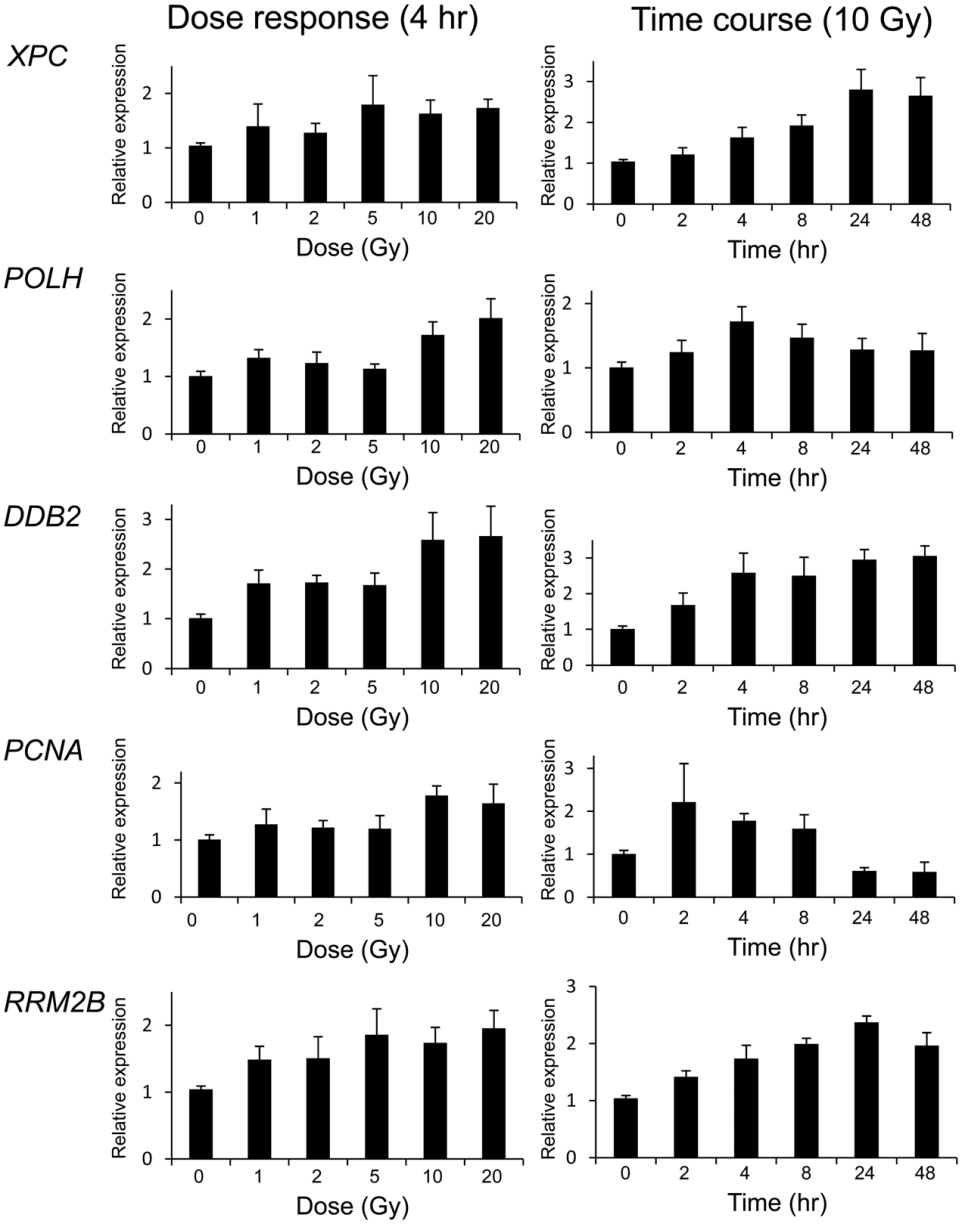
Expression levels of the top five DNA repair genes as determined by qRT-PCR in fibroblasts over different IR doses and times. Relative gene expression is shown for samples that were either sham irradiated or irradiated with 1 Gy, 2 Gy, 5 Gy, 10 Gy or 20 Gy of radiation at 4 hours post-IR, or sham irradiated or irradiated with 10 Gy at 2 h, 4 h, 8 h, 24 h or 48 h post-IR.

The radiation response between the two cell types were compared and found to show a similar response for most DNA repair genes, however, the LCL response generally had a larger induction at the 4 hour time point compared to the fibroblast cell lines. This is especially evident for *PCNA* and *RRM2B* genes, where there is more than a two-fold difference between the cell types (Figures 1,2,3; Tables 1 and 2). Dose response and time course experiments were consistent with this observation (Figures 4,5,6). However, microarray expression levels (Figures 1 and 2) and additional qRT-PCR data (Figure S3) indicate that the initial levels of transcripts for *XPC* and *RRM2B* are higher in the fibroblasts compared to the LCLs (relative to the control gene PGK).

### DNA Repair Gene Expression dose Response and Time Course

The effect of dose on DNA repair gene expression response was investigated using whole genome exon arrays at four hours post-treatment using both LCLs and primary fibroblasts (Figure 4). Some genes such as *POLH*, had a robust response to a relatively low dose of 1 Gy, and then consistent but smaller incremental responses to IR with increasing doses of radiation up to 20 Gy (Figure 4A). *DDB2* showed a similar pattern to *POLH* in LCLs but the fibroblast group showed a more gradual transcript level increase with dose (Figures 4C and 4E). These results were consistent with qRT-PCR expression analysis (Figures 5 and 6). Other genes such as *XPC*, *PCNA* and *RRM2B* showed a dose dependent response in both LCLs and fibroblasts although these responses were generally less in fibroblasts (Figures 5 and 6). An additional set of 12 arrays of the fibroblast samples isolated 4 hr after 2 Gy IR were also performed, and although the inductions were generally less, the results were consistent with the 10 Gy responses in this cell type (data not shown).

DNA repair gene expression across different time points was investigated up to 48 hrs post-irradiation in both LCLs and fibroblasts treated with 10 Gy. Early robust responses were identified 2 hours post-IR in LCLs for *POLH* and *DDB2* showing peak expression 8 hours after treatment, followed by a decrease after 24 hours, and the levels of transcripts were still above basal expression levels at 48 hours (Figure 4). Temporal patterns of gene modulation were validated using qRT-PCR for these genes and for *XPC*, *PCNA* and *RRM2B* (Figure 5). The results were consistent between exon array and qRT-PCR data. The peak expression levels in the fibroblast cells were different from the LCLs for *XPC* and *RRM2B*. In the LCLs, expression levels were highest at 8 hours post-irradiation and decreased by 48 hours whereas in the fibroblast cells the expression did not peak until 24 hours and was still elevated at 48 hours (Figures 5 and 6). The *DDB2* expression levels in the fibroblast cells differed slightly to the levels in LCLs. The expression levels in both cell types peaked at 8 hours. However, in the LCLs, the levels start dropping 24 hours post irradiation but they remain high in the fibroblasts up to 48 hours post irradiation (Figures 3, 4, 5 and 6). For *POLH*, and *PCNA*, peak expression levels were similar in the LCLs and fibroblasts (Figures 5 and 6).

### Alternative Transcription in DNA Repair Genes

**Figure 7:**
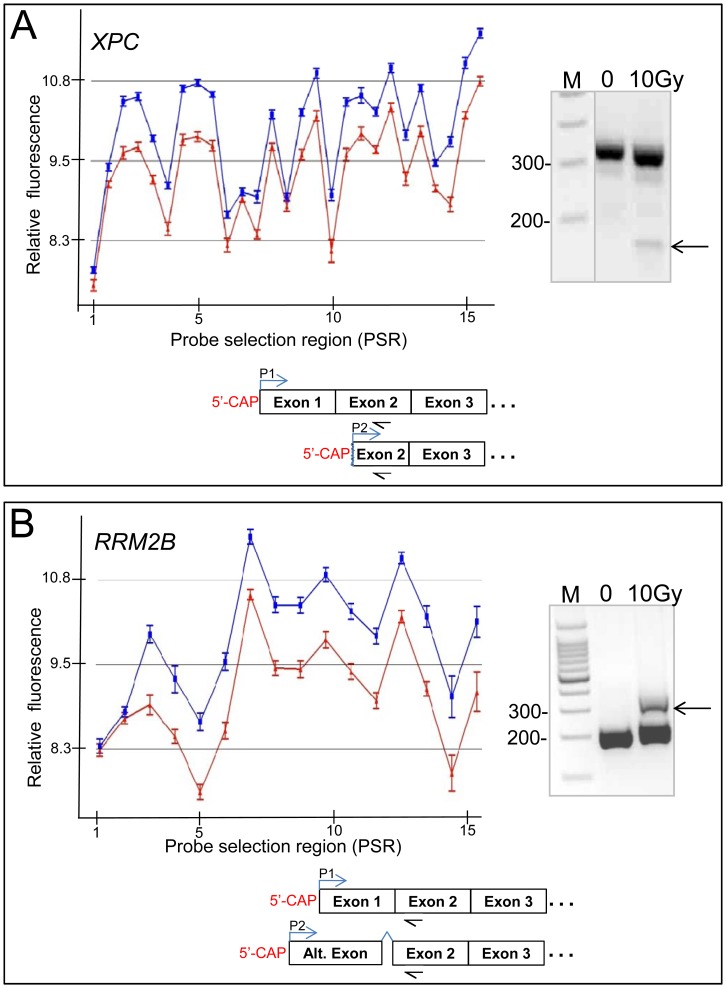
Alternative transcripts in DNA repair genes are induced by IR. Alternative transcripts in the DNA repair genes, XPC (A) and RRM2B (B) in response to IR are shown. Graph axes are as in figure 1; PCR products from 5′-RML-RACE were run on a 2% agarose gel. An arrow indicates the amplicon from the alternative initiated transcript that was sequenced (gel picture to the right of the panel; Figure S4). Diagrams of the predominant transcripts (initiating by P1) and the alternatively initiated (P2) transcripts after IR are shown below. Primer locations for 5′ RLM-RACE are indicated below exon 2 (arrow pointing to the left).

The comprehensive exon coverage of this array platform enabled us to investigate alternative transcription products. Certain DNA repair genes showed variations across the gene in that some PSRs showed different levels of expression changes after treatment with IR. This suggests induction or changes in levels of alternative transcripts following irradiation. For example, *PCNA* showed features of alternative termination (Figure 1D). Other genes, such as *XPC* and *RRM2B* showed a robust level of expression in all but the first two 5′ PSRs (Figure 7). To confirm the use of an alternative transcriptional initiation sites we performed 5′-RLM-RACE using RNA transcripts from LCLs for *XPC* and *RRM2B*. A shorter *XPC* transcript was identified after IR. Sequencing (Figure S4) of PCR amplified amplicon (150 bp; Figure 7A) confirmed that the start site of this shorter *XPC* transcript occurred within exon two, 261 bp 3′ of the full length transcript’s initiation site (NCBI sequence NM_004628). This transcript is similar to the NCBI *XPC* sequence X65024 which has a transcription start site 265 bp 3′ of the full length transcript. The predicted isoform (NCBI protein id: CAA46158.1) encodes for a protein missing 117 amino acids from the N-terminus of the full length XPC protein isoform. The 5′-RLM-RACE PCR data suggest this radiation induced shorter transcript is probably a minor product (Figure 7A) and the full length transcript is also radiation induced. Prediction of Protein Sorting Signals and Localization Sites in Amino Acid Sequences (PSORT) analysis (http://psort.hgc.jp) of the predicted proteins suggests that the missing N-terminus region contained several coiled-coil regions (Lupas’s algorithm).

A longer alternative transcript was identified in *RRM2B* after irradiation as depicted by a 334 bp PCR amplicon (Figure 7B) which was identified by sequencing (Figure S4) to be a transcript with a start site at nucleotide 35 of the published variant 2 transcript sequence (NCBI: NM_001172477). The predicted isoform 2 would be missing the first 16 amino acids of the predominant isoform 1 protein and would have an additional 88 unique amino acids at the N-terminus (NCBI protein NP 001165948). The exon array expression profile from the fibroblasts also supports the expression of this longer variant 2 transcript after IR (Figure 1E). PSORT analysis (κ-NN prediction) predicts that the isoform 1 has a 61 percent chance to be directed towards cytoplasmic localization with a 26 percent chance for nuclear localization and a 9 percent chance for mitochondrial localization. However, isoform 2 is predicted to have a 43 percent chance for localization to the mitochondria, with only a 35 and 22 percent chance for cytoplasmic or nuclear localization, respectively.

## Discussion

These investigations have provided a comprehensive investigation of the transcription response of DNA repair genes to DNA damage caused by IR on the exon level in two divergent cell lineages. Little is known about alternative transcripts which are modulated by IR and this aspect of transcription has been addressed. Thirty DNA repair genes were identified as radiation responsive at the transcript level. Many of these DNA repair genes did have relatively low levels of transcriptional modulation at the early time point of 4 hours. Some of these genes have not previously reported to be responsive to IR. The small changes for some of these transcripts may be why other less sensitive analyses failed to detect them. Some genes showed modest lower expression levels after IR. The products of the down-regulated genes may be required for cell cycle progression and replication where proofreading and other DNA repair activity is needed during these normal cell processes, and may be a reflection of the IR induced cell cycle arrest. IR produces free radicals resulting in oxidative stress [31]. Repair of oxidative DNA lesions involve the BER and NER DNA repair pathways [32]. Our study has shown several NER pathway genes (*XPC*, *DDB2*, and *LIG1)* and a BER pathway gene (*MBD4)* are modulated by IR (Tables 1 and 2).

Effects of dose and time after IR on the DNA repair gene transcription was also investigated to gain further insights into the kinetics of the IR-induced DNA repair gene transcriptional response. The dose kinetics of the DNA repair genes in most cases showed increased gene expression at the higher doses, but commonly reached a maximal level earlier than the maximal dose indicating a plateau effect. Many of the genes also showed a distinct maximal response prior to 48 hours post-IR. The variation in response kinetics can reveal some insight into the responses such as those genes that may be coordinately regulated in response to p53 such as *XPC*, *RRM2B*, *DDB2* and *POLH.*

Alternative transcripts of *RRM2B* and *XPC* in response to damage were also identified. In both these genes, the 5′UTR and N-terminus of the predicted protein isoforms differ from that of the predominant transcript. The *RRM2B* gene product (p53R2) is a p53 regulated ribonuclease reductase small subunit which supplies deoxyribonucleoside triphosphates to sites of DNA damage during DNA repair [33]. p53R2 is known to be induced by DNA damaging agents such as IR and is involved in the p53 regulated cell cycle arrest checkpoint [33]. p53R2 is usually distributed throughout the cytoplasm, but it accumulates in the nucleus following DNA damage induced by UV irradiation [34]. The first 113 amino acids of p53R2 of the most highly expressed transcript (isoform 1) are reported to be critical for the interaction with N-terminus of the cell cycle regulator, p21 [34]. When p53R2 and p21 are located in the nucleus, the interaction between the two proteins decreases and there is a concurrent increase in ribonuclease activity [34]. Although isoform 2 has been reported to the NCBI data system (NCBI: NP 001165948), no reference to its function or specific expression has been described. The p53R2 isoform 2 is missing the first 16 amino acids present in isoform 1 and has an additional 88 unique amino acids at its N-terminus. As the first 113 amino acids of isoform 1 are involved in binding p21, this interaction is probably altered in the IR induced isoform. Another possible aspect of isoform 2 is that it may have altered cellular localization. The radiation responsive gene product, FBXW7, is an example of a protein that has isoforms that differ in their N-terminus which show different subcellular localization [35]. It is possible that the damage induced p53R2 isoform 2 may localize to DNA containing organelles such as the mitochondria and nucleus rather than the cytoplasm. The difference in the N-terminus may also affect the association with p21.

We confirmed that *XPC* is induced by IR at the transcription level, which has been consistently observed in several studies [18], [30]. XPC is involved in DNA damage sensing for NER. XPC recognizes various forms of DNA damage that result in distortion of the DNA helix resulting in short sections of single stranded DNA [36], [37]. XPC binds to the undamaged nucleotides identifying the template strand for DNA repair, then recruits transcription factor IIH (TFIIH) complex [38]. Recently, XPC has been shown to enhance UV-induced apoptosis by directly binding to the promoter of caspase-2S gene preventing its transcription [39]. The IR induced *XPC* transcript that we identified using RACE (Figure 7) is a minor product compared to the normally expressed XPC transcript and the resulting predicted protein product, would be missing the first 117 amino acids. XPC protein N-terminus region contains several coiled-coil regions but has not been well characterized. However aa 156–325 have been shown to interact with DNA and XPA [40]. The C-terminus of XPC (aa 492–940) binds to DNA and also interacts with various proteins including RAD23B, CENTN2, and TFIIH. A mRNA transcript (NCBI: X65024) similar to that identified in this study was previously identified in a cDNA clone (XPCC). The cDNA clone increased UV-radiation survival when transfected into a XPC derived cell line [41] indicating that this transcript is capable of producing a functional protein. The functional difference between the two XPC isoforms is presently unknown.

The 5′UTR and N-terminus sequences are known to be responsible for the efficiency of translation, stability, subcellular localisation, cellular and tissue specific expression. It is known that N-terminus sequences often define protein half-life [42]. This may be a crucial mechanism to provide specific function to repair in response to DNA damage and crosstalk with pathways regulating cell cycle and apoptosis. The importance of the induction of alternative transcripts in the DNA damage response has been described for several genes including *MDM2* and *CDKN1A*. DNA damage has been shown to induce isoforms, in a p-53 dependent manner, in both MDM2 [43], [44], [45], [46] and *CDKN1A* [47]. In the case of *MDM2*, radiation induces a shorter transcript by using a p53-regulated alternative start site. p53 plays a major role in induction of IR-induced expression of *XPC*, *PCNA, RRM2B*, *DDB2* and *POLH* [48], [49], [50], [51] and may also be involved in the induction of the alternative transcripts induced by IR detected in our studies.

The responses to IR between two cell lineages, LCLs and fibroblasts, were compared. Although much of the DNA repair transcription response to IR was similar, it was not identical. The different levels of initial transcript, induction times, and levels after irradiation shown for some specific genes may contribute to the unique cellular responses attributed to these two different cell types. We have found a number of NER repair genes (e.g. *XPC, POLH (XPV), DDB2 (XPE)* and other non-dsb repair genes (e.g. *PCNA, RRM2B*) to be induced by IR. The induction of transcription for several NER genes by IR (Table 1) might not only play a role in DNA damage repair but also in a cell fate decision. In fact, studies have shown that two NER proteins, XPC and POLH (XPV), enhance apoptosis [39], [48]. The fact that the expression of a number of NER proteins are up-regulated, and induction of alternative transcripts is observed along with the changes in expression of several apoptosis mediators ([19] and unpublished results) to a higher extent in the radiosensitive LCLs, brings out the potential role for these NER associated proteins in apoptosis. It may have been expected that DNA dsb repair genes such as those found in NHEJ and HR would be induced after IR given their critical nature, but perhaps the numbers of these types of breaks that are normally encountered did not warrant the evolutionary development of regulation of these repair genes at the transcription level in these time frames and regulation at the protein level is a more efficient mechanism for immediate DNA dsb repair. Furthermore, the transcriptional responses for many DNA repair genes may be too subtle for detection.

### Conclusion

This investigation provides a comprehensive study of DNA repair gene expression dynamics at the exon level in response to the DNA damaging agent, IR, in human cells. These data provide IR response kinetics covering a range of doses and times, probing at the exon level. Novel expression of alternative transcripts has been examined and DNA repair genes that utilize a mechanism of alternative transcription initiation following IR have been identified.

## Supporting Information

Figure S1 Modulation of DNA repair genes at the exon level 4 hours after treatment with 10 Gy IR in lymphoblast cell lines. PSR expression levels are plotted for each of the DNA repair genes with a p-value of <0.05 using Partek Genomics Suite statistical package. Relative fluorescence (y-axis; log base 2) is plotted for each PSR (x-axis). Core PSRs are labelled below the graphs. Samples were either sham irradiated (red) or irradiated (blue) with 10 Gy from a ^137^Cs source. Error bars = SEM (n = 12).
(PDF)

Figure S2 Modulation of DNA repair genes at the exon level 4 hours after treatment with 10 Gy IR in fibroblast cells. PSR expression levels are plotted for each of the DNA repair genes with a p-value of <0.05 using Partek Genomics Suite statistical package. Relative fluorescence (y-axis; log base 2) is plotted for each PSR (x-axis). Core PSRs are labelled below the graphs. Samples were either sham irradiated (red) or irradiated (blue) with 10 Gy from a ^137^Cs source. Error bars = SEM (n = 12).
(PDF)

Figure S3 Real-time PCR comparison of DNA repair gene expression between LCLs and fibroblasts. Initial levels of transcripts are significantly higher in fibroblasts (FB) for *XPC* and *RRM2B* relative to LCLs, but after IR induction, gene expression levels are not significantly different. Values have been normalized to *PGK* gene expression. The p-values for differences of initial levels between LCL and fibroblast samples are as follows: *XPC*: p = 0.0025 and for *RRM2B*: p = 0.0030.
(PPTX)

Figure S4 Sequence analysis of the 5′RACE amplicons for *RRM2B* (A) and *XPC* (B). Sequences homologous to the primers used for their amplifcation are indicated by long horizontal arrows below the sequence. The adaptor sequences are shown in capital letters. The vertical arrows indicate the start site for the capped mRNA transcript. This is nucleotide 35 of NCBI *RRM2B* sequence NM_001172477 (A), and at nucleotide 261 of NCBI *XPC* sequence NM_004628 (B).
(PPTX)

Table S1 PCR primer sequences.
(XLSX)
