# Supplementary figures and images for: DNA Repair Genes: Alternative Transcription and Gene Expression at the Exon Level in Response to the DNA Damaging Agent, Ionizing Radiation
Source: PLoS One. 2012 Dec 28;7(12):e53358. doi: 10.1371/journal.pone.0053358 (PMC3532210; doi:10.1371/journal.pone.0053358)

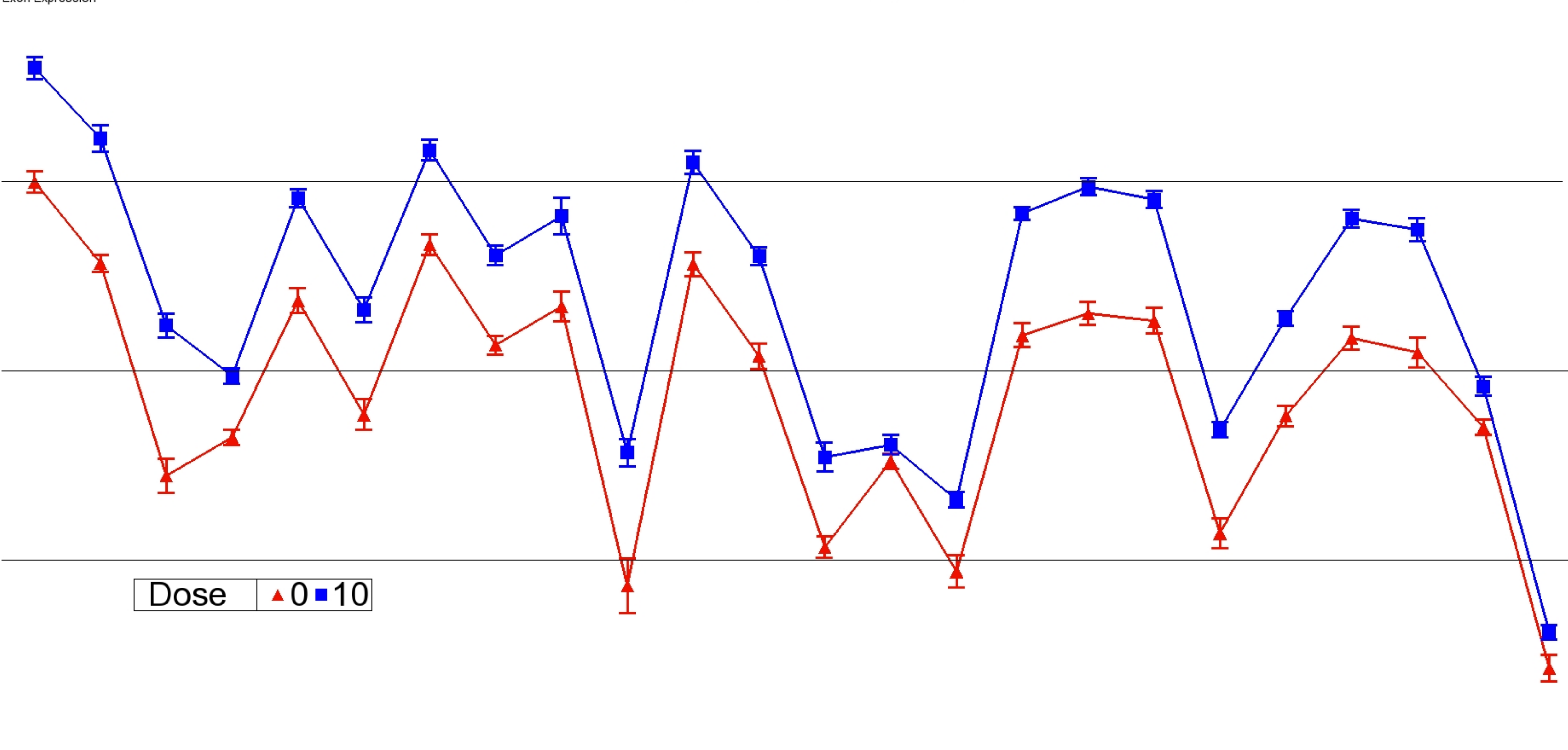

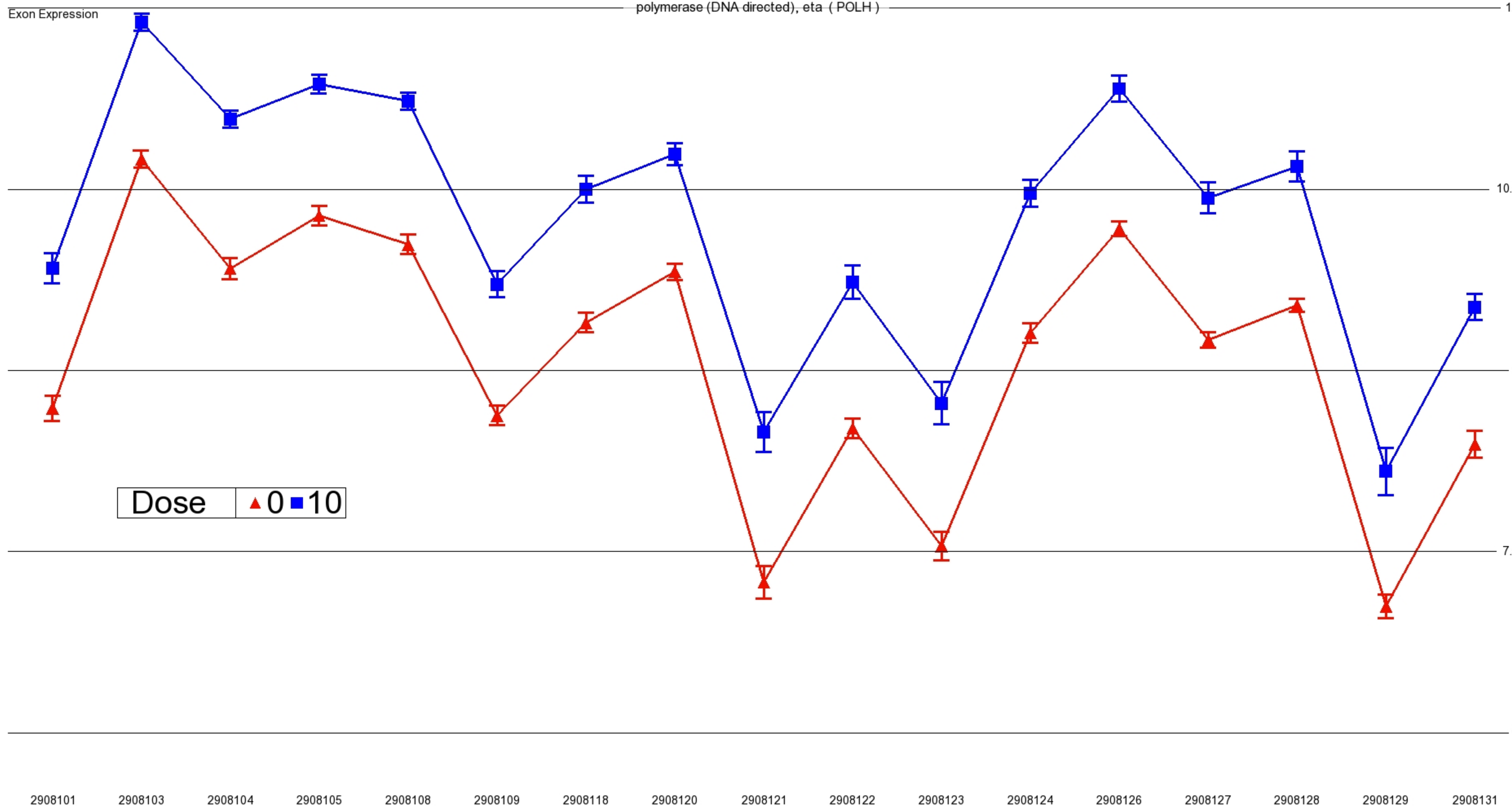

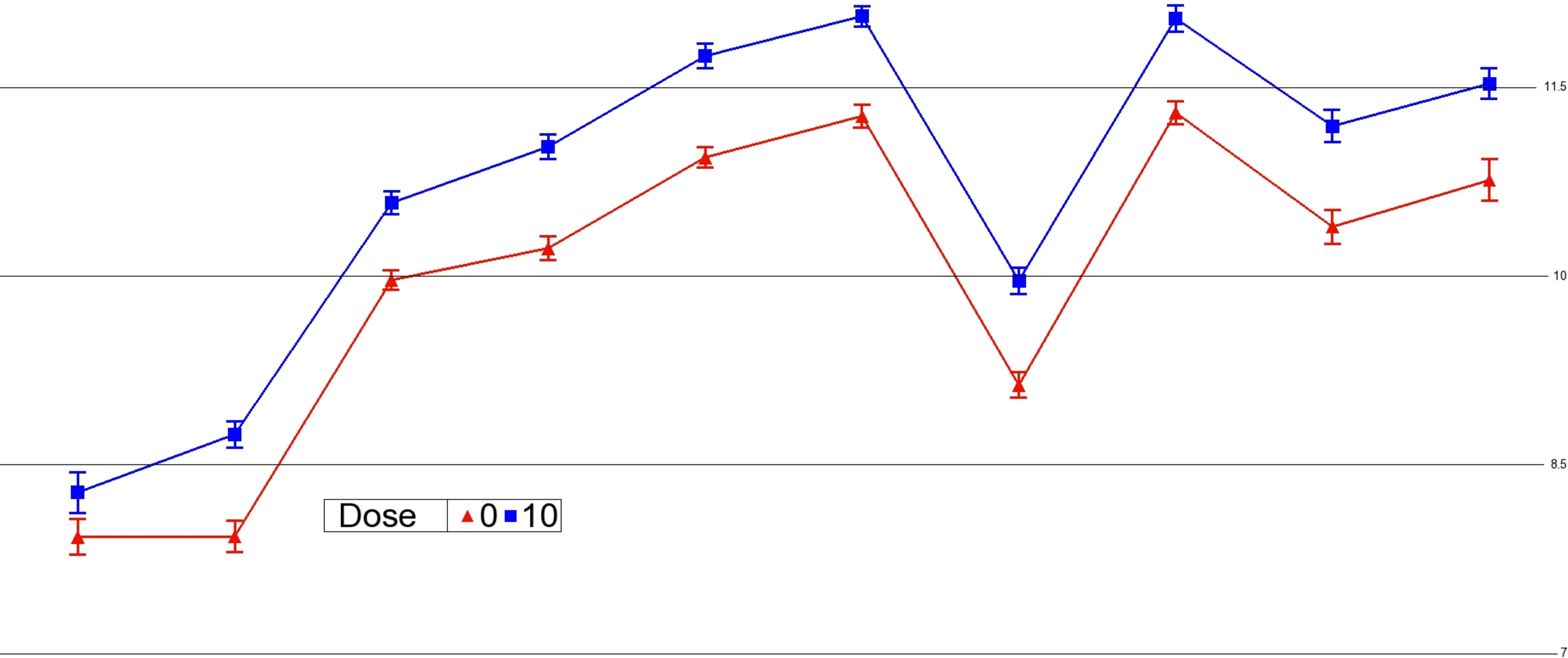

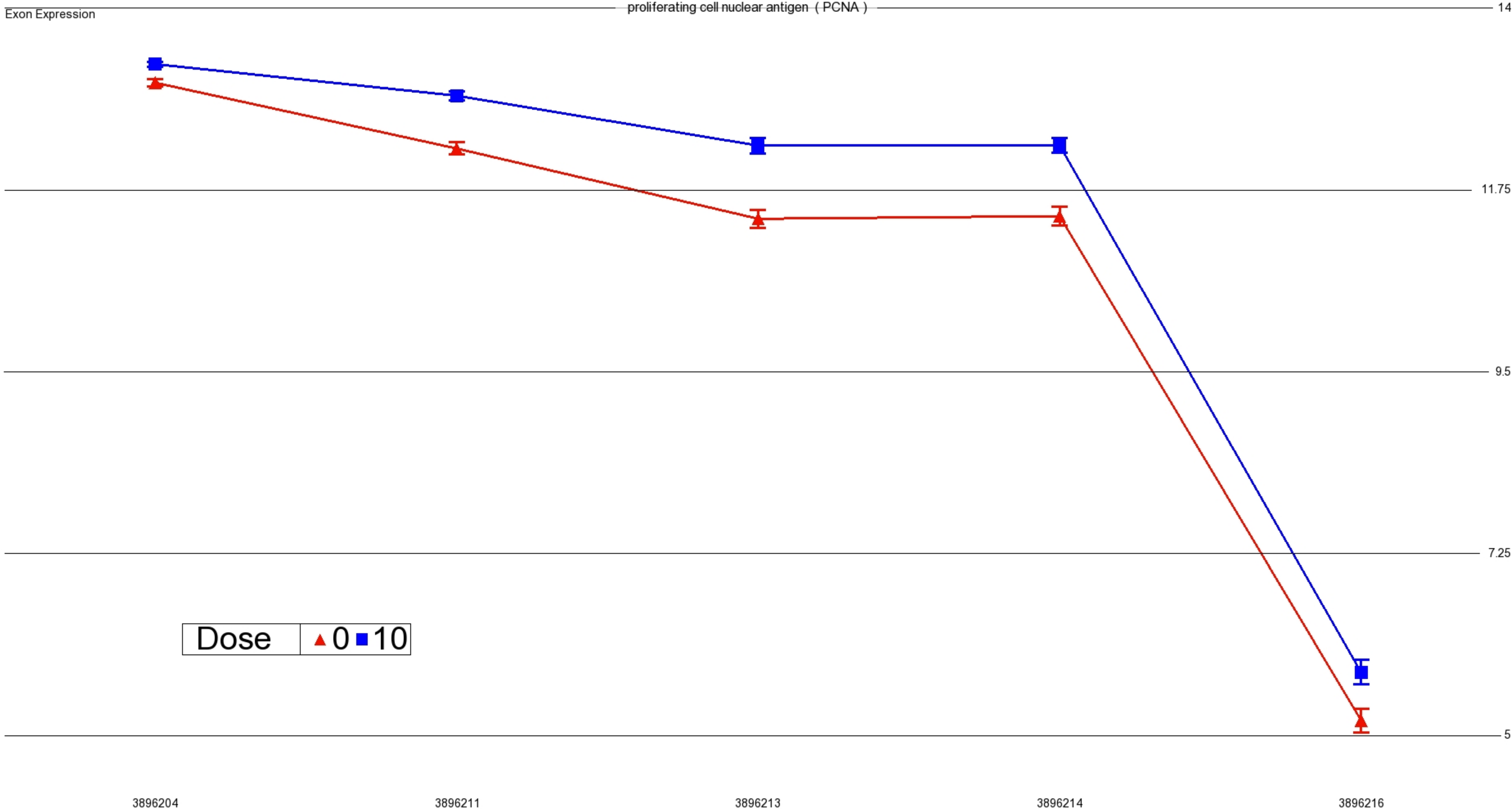

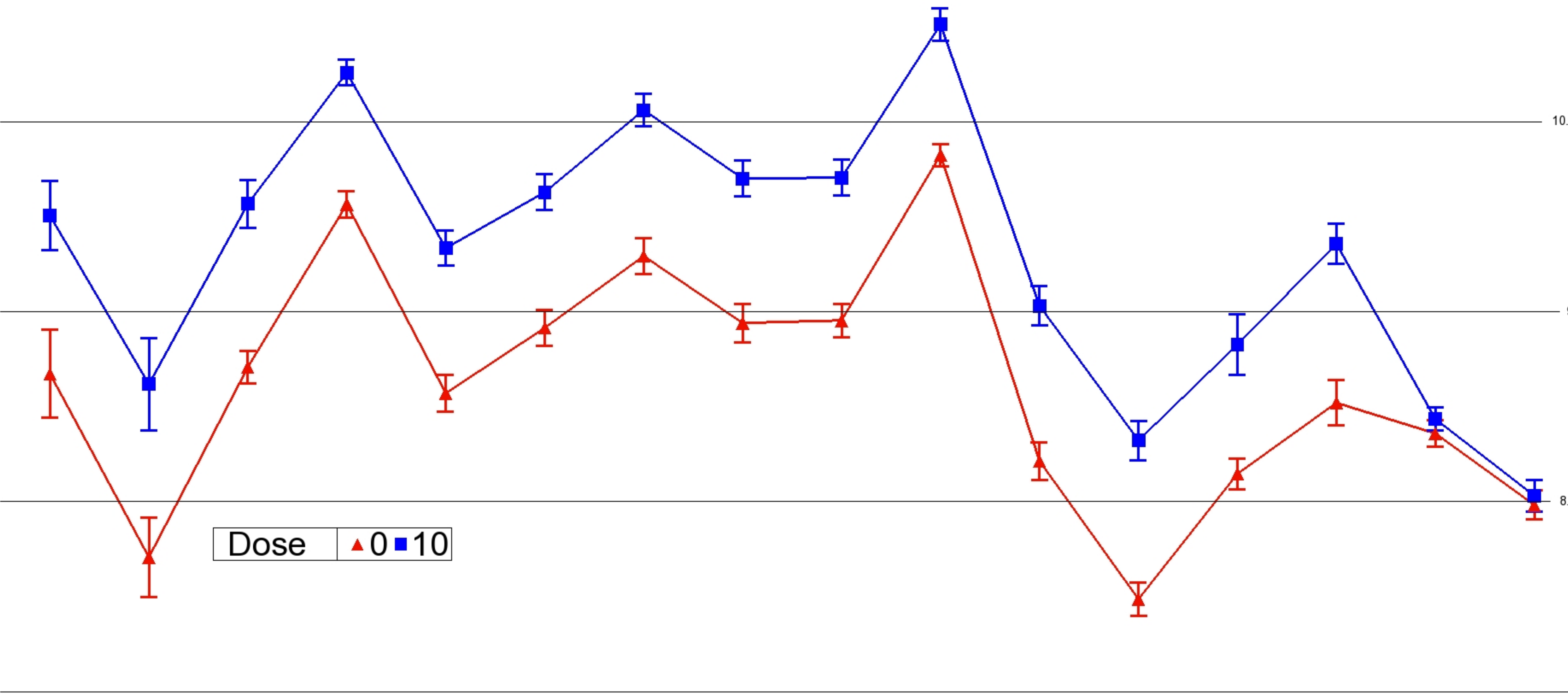

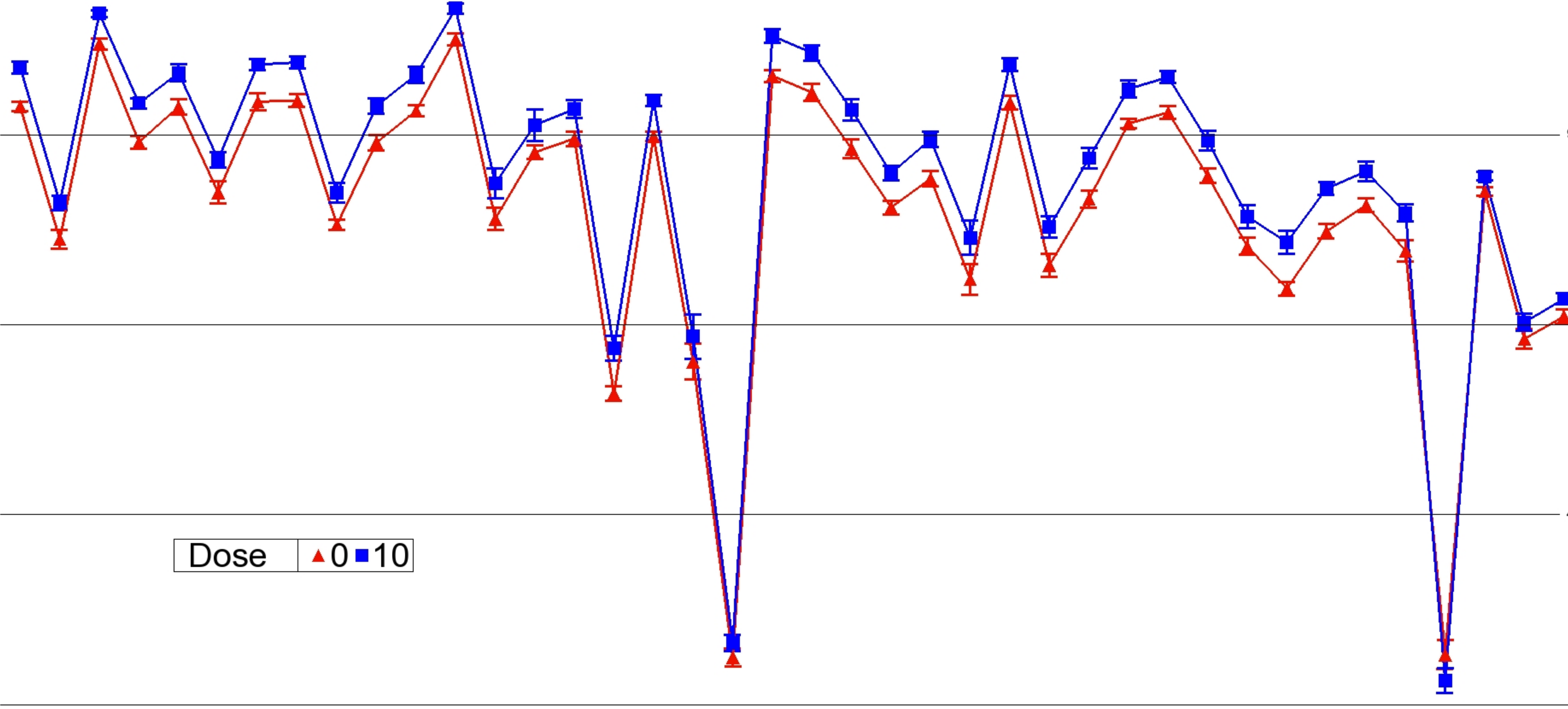

Dose

▲ 0 ■ 10

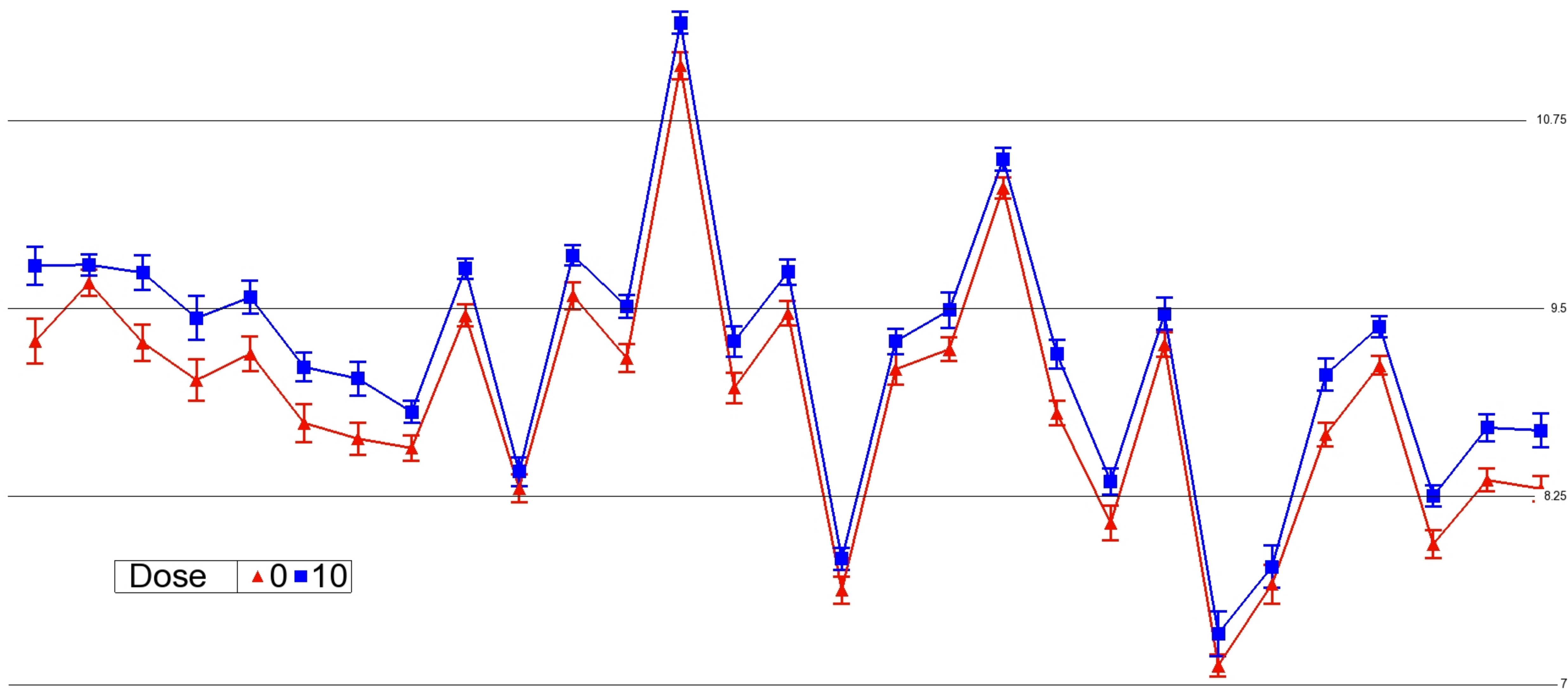

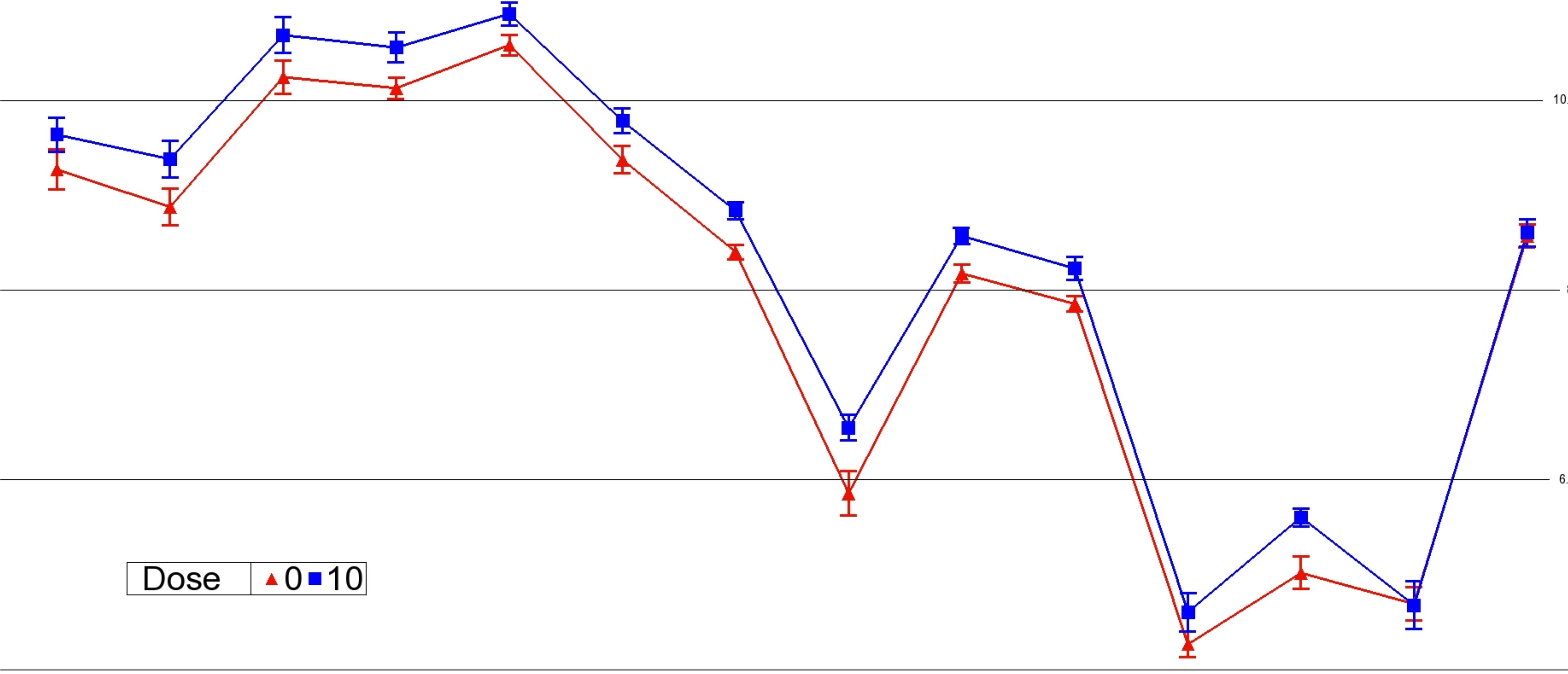

Dose    ▲ 0    ■ 10

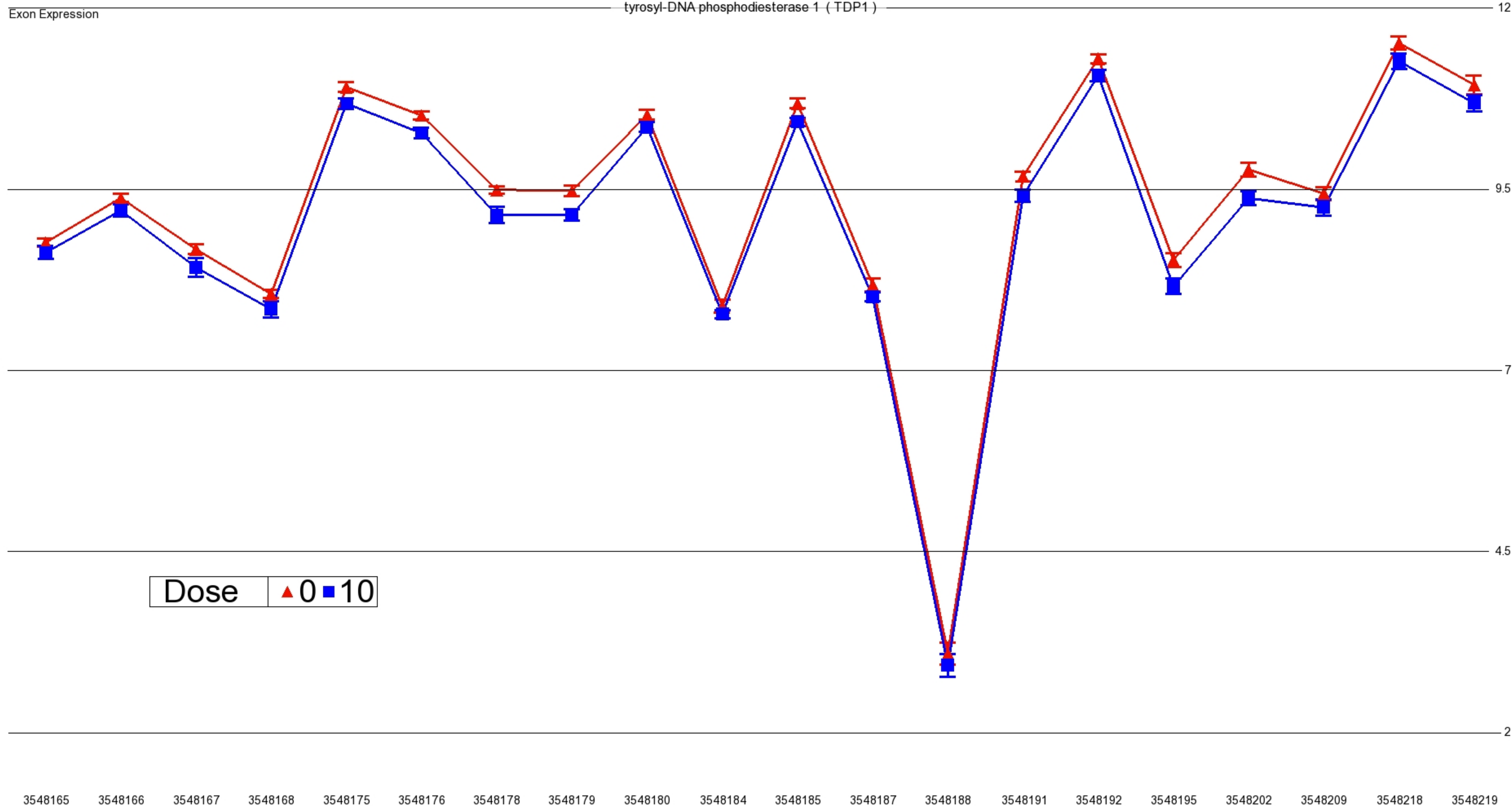

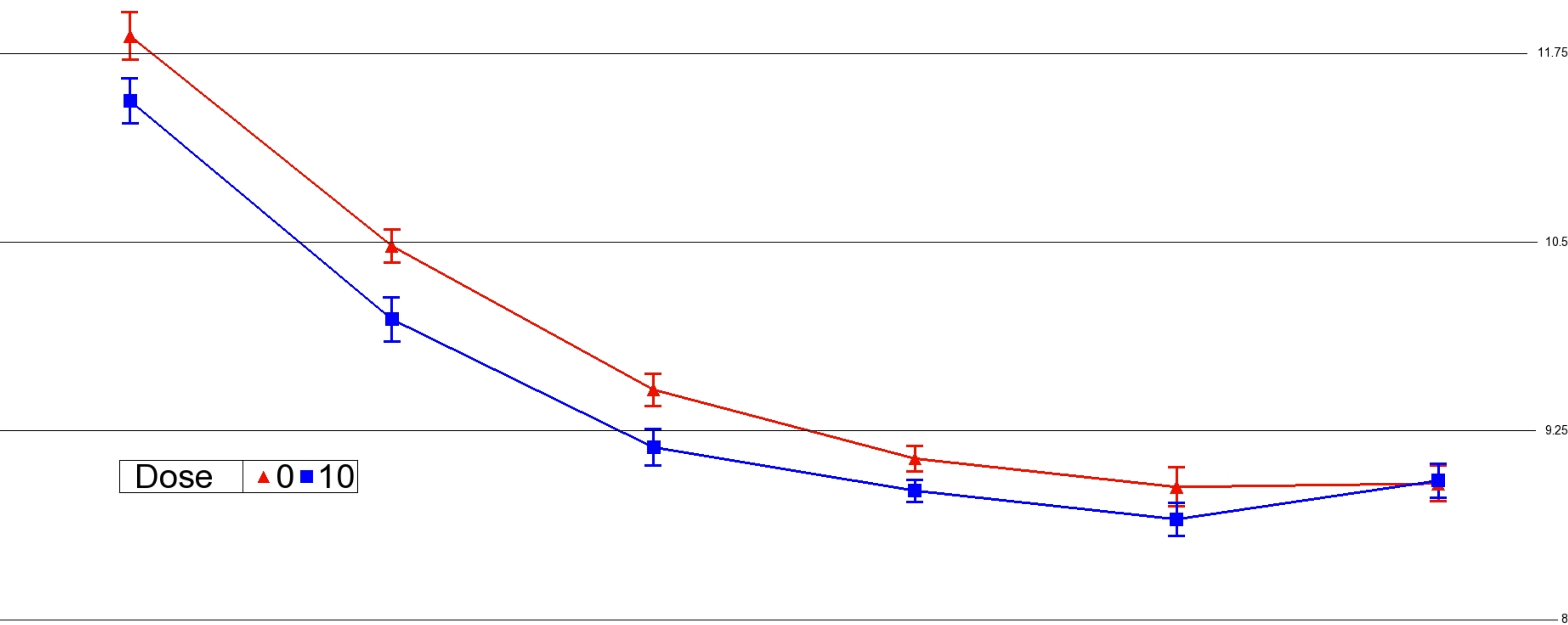

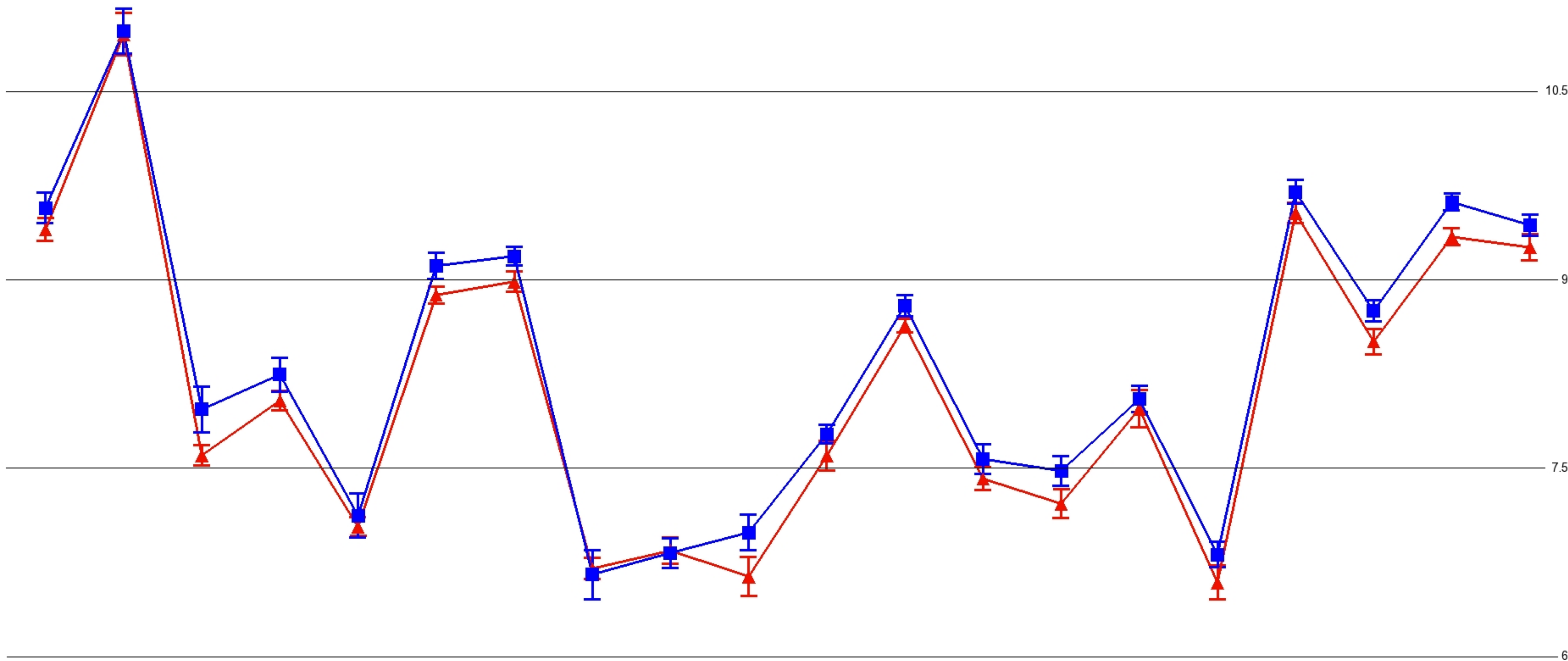

Dose

▲ 0 ■ 10

3817502

3817503

3817505

3817507

3817508

3817509

3817510

3817513

3817514

3817519

3817520

3817521

3817523

3817524

3817525

3817526

3817530

3817531

3817532

3817534

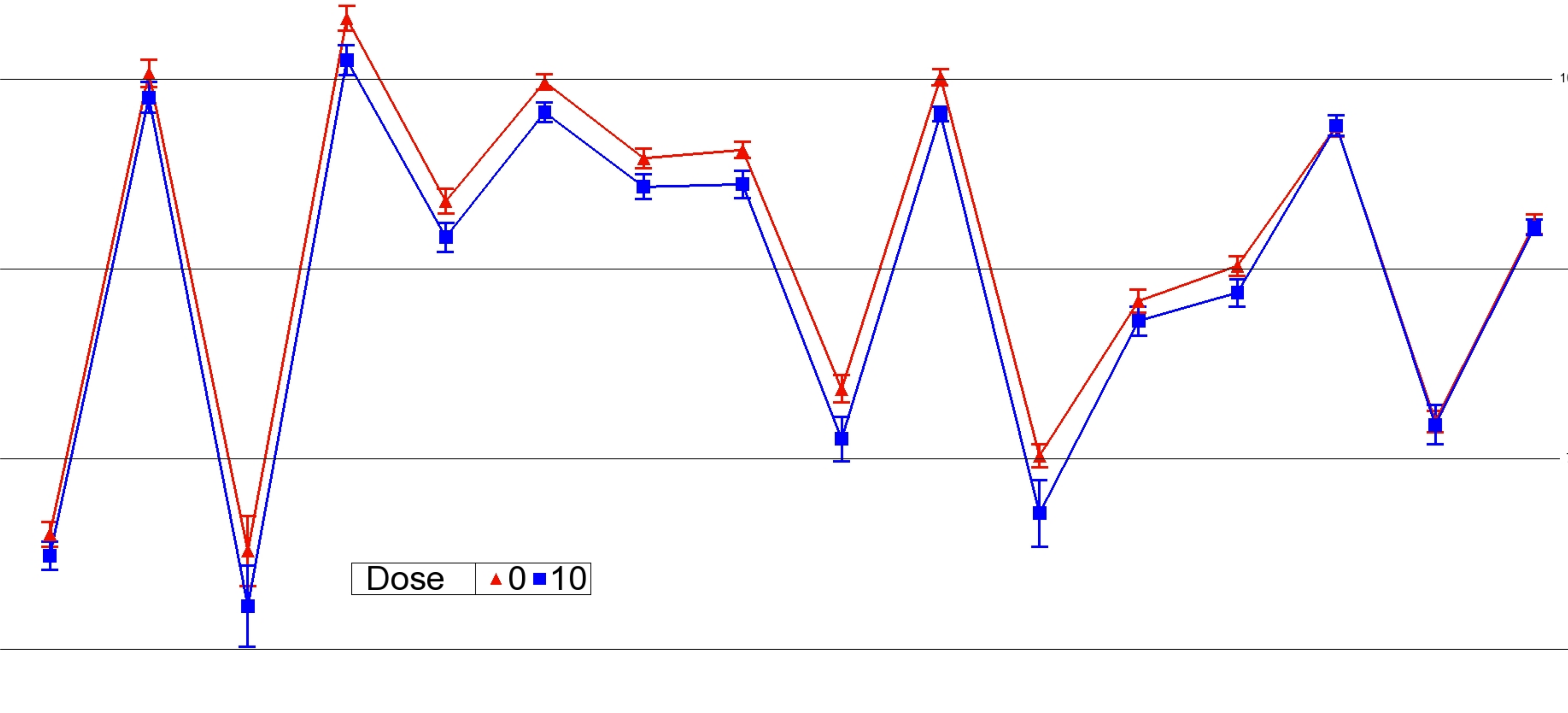

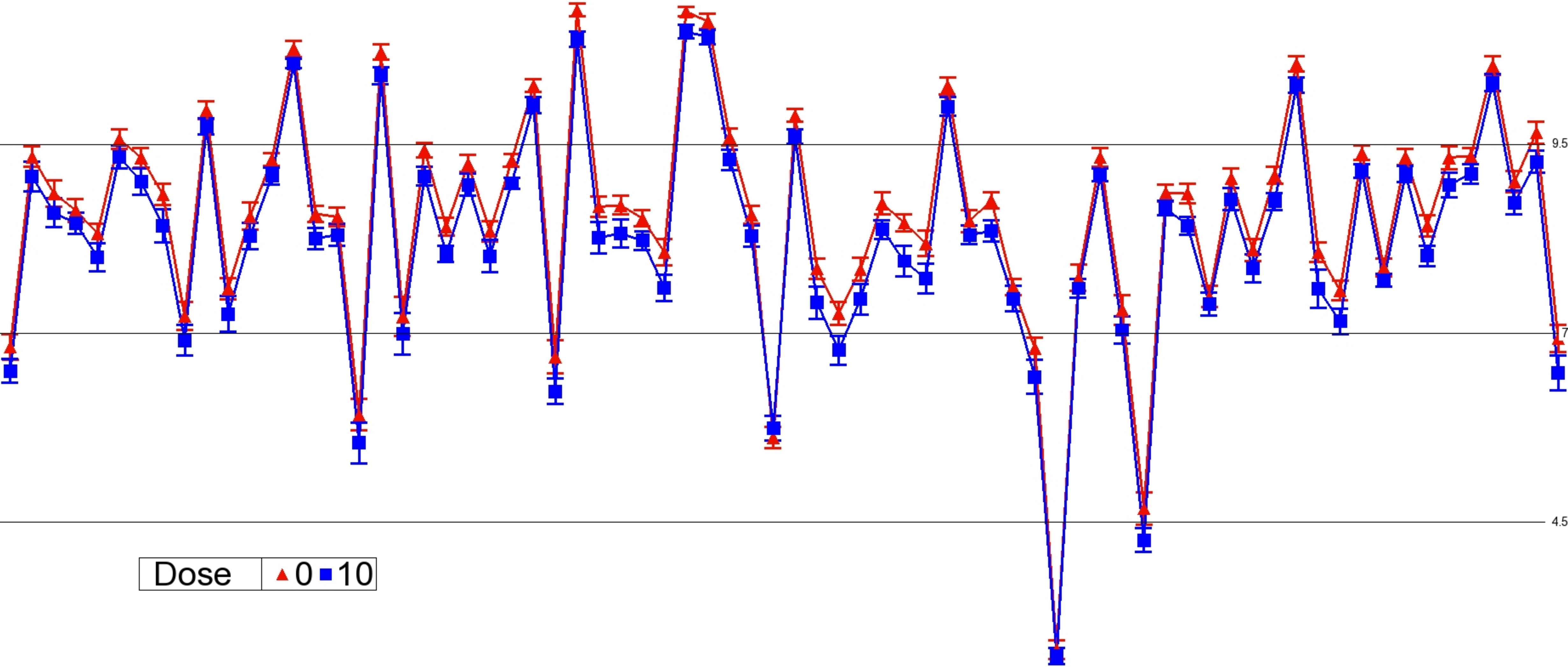

Dose    ▲ 0    ■ 10

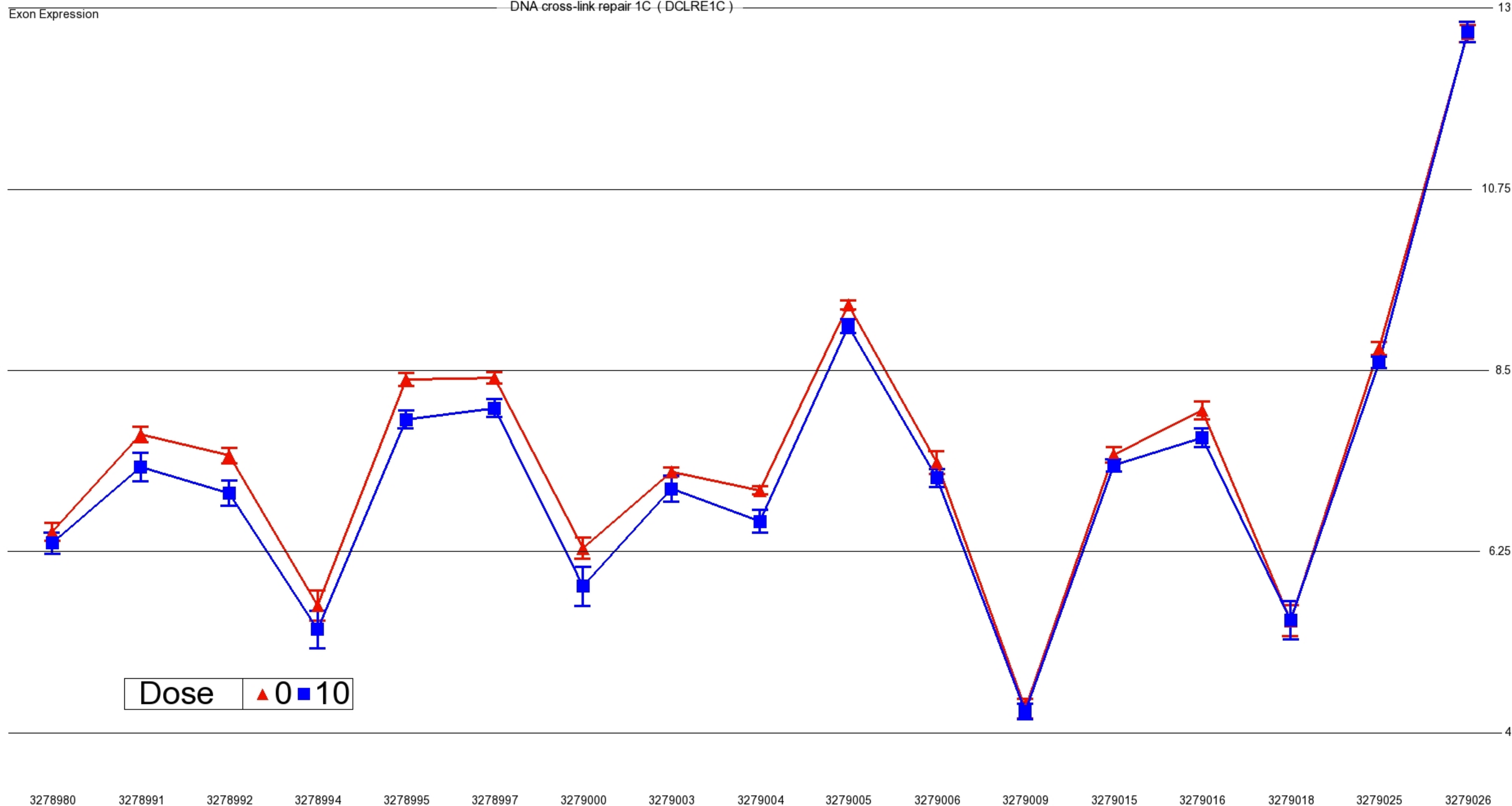

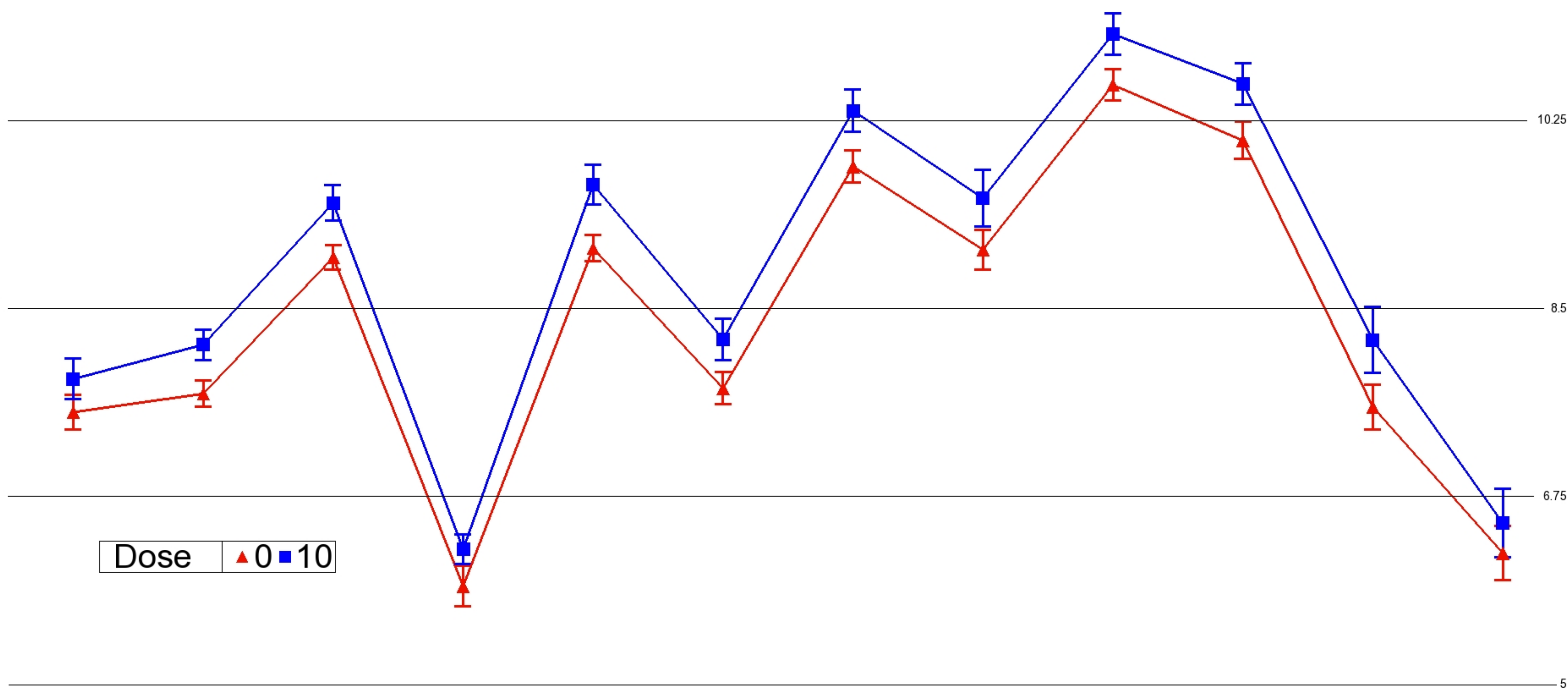

Dose    ▲ 0    ■ 10

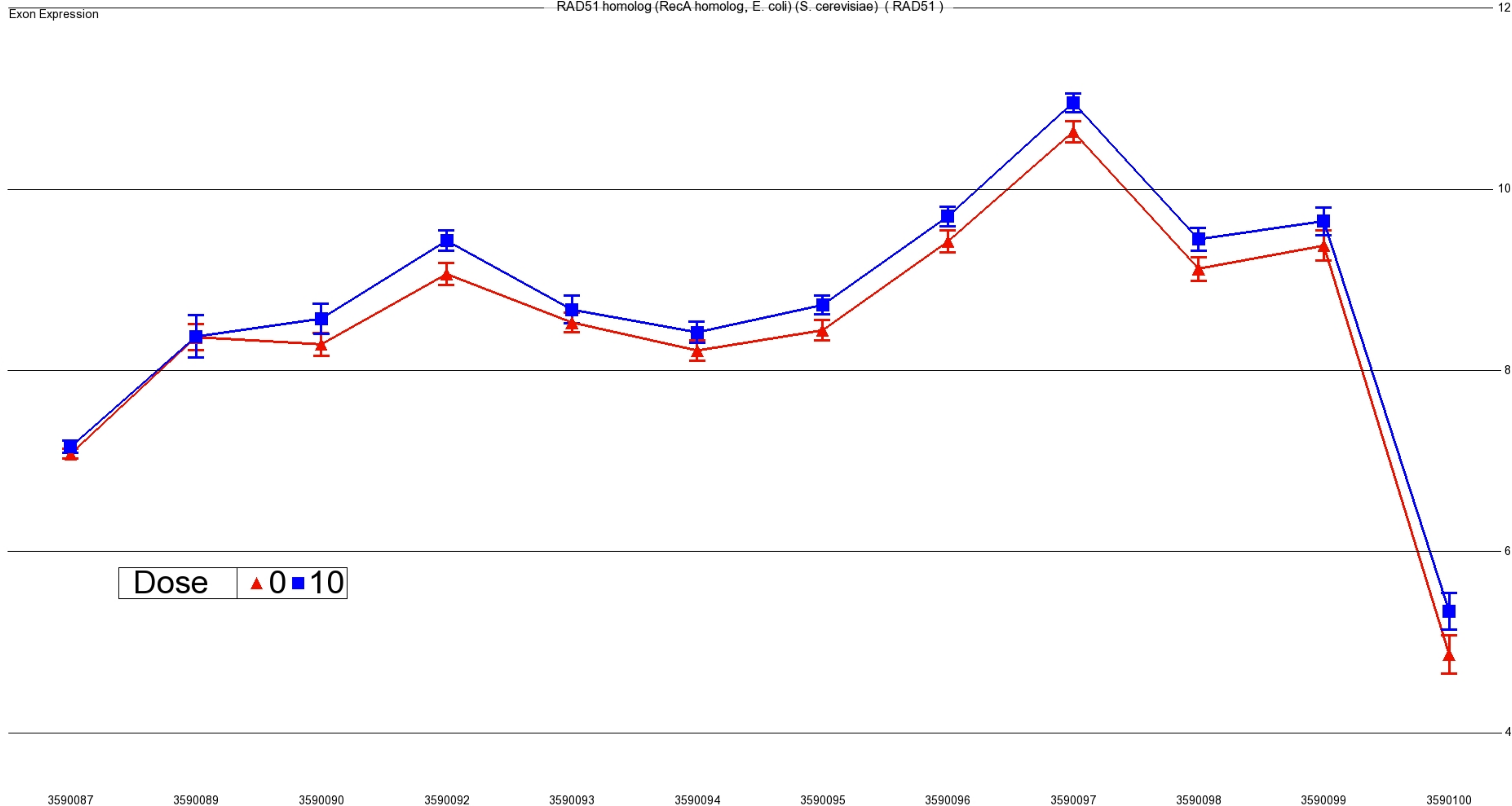

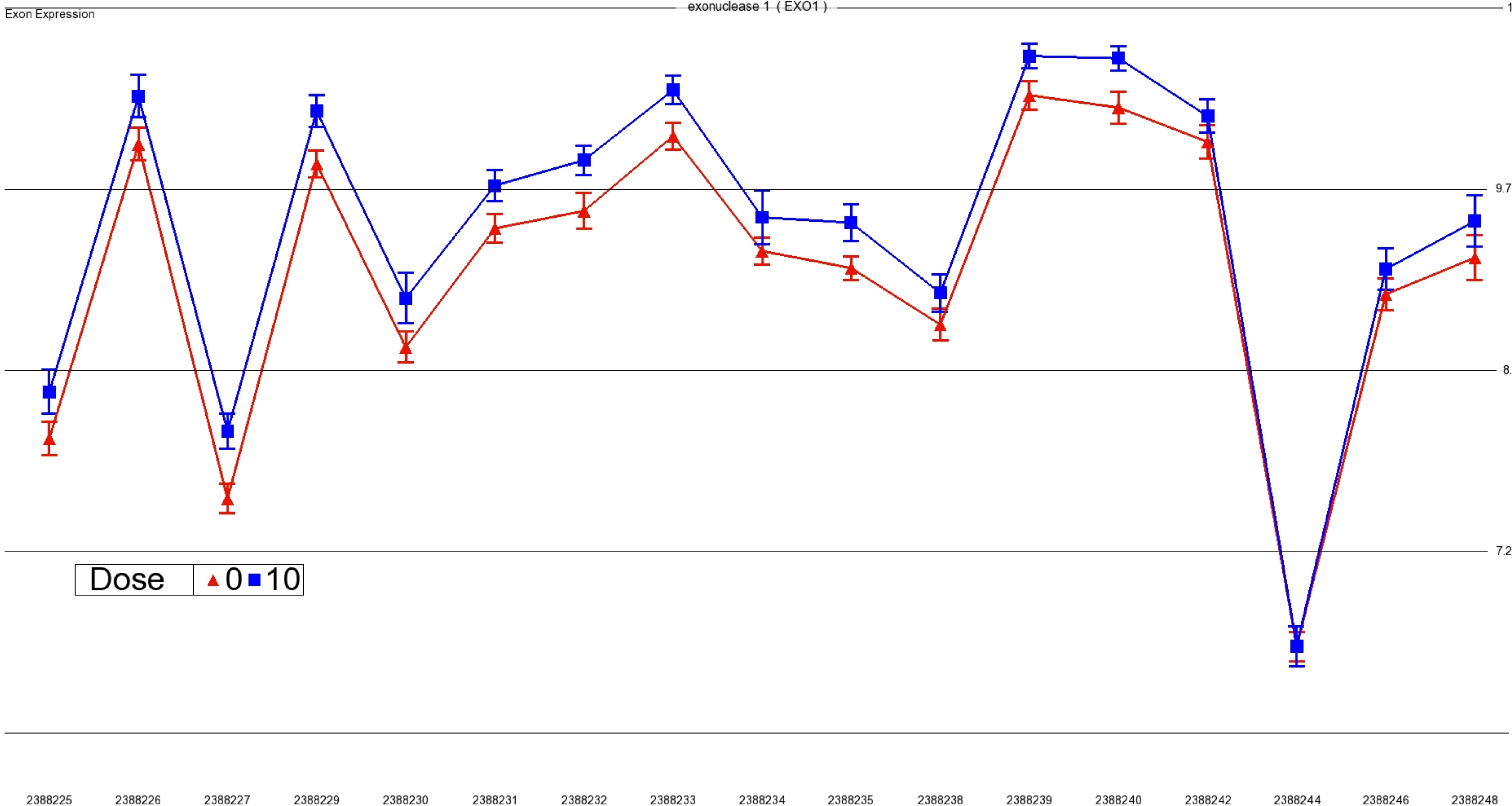

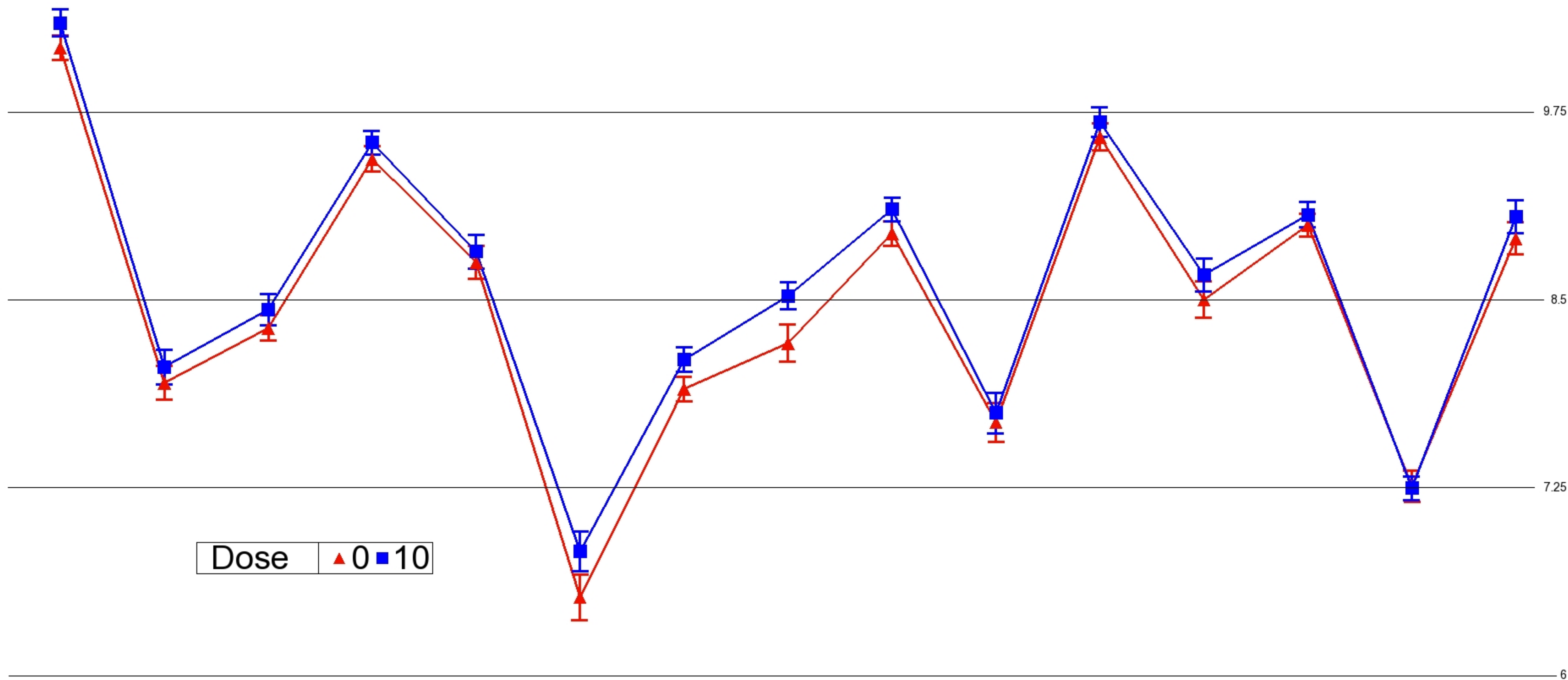

Dose

▲ 0 ■ 10

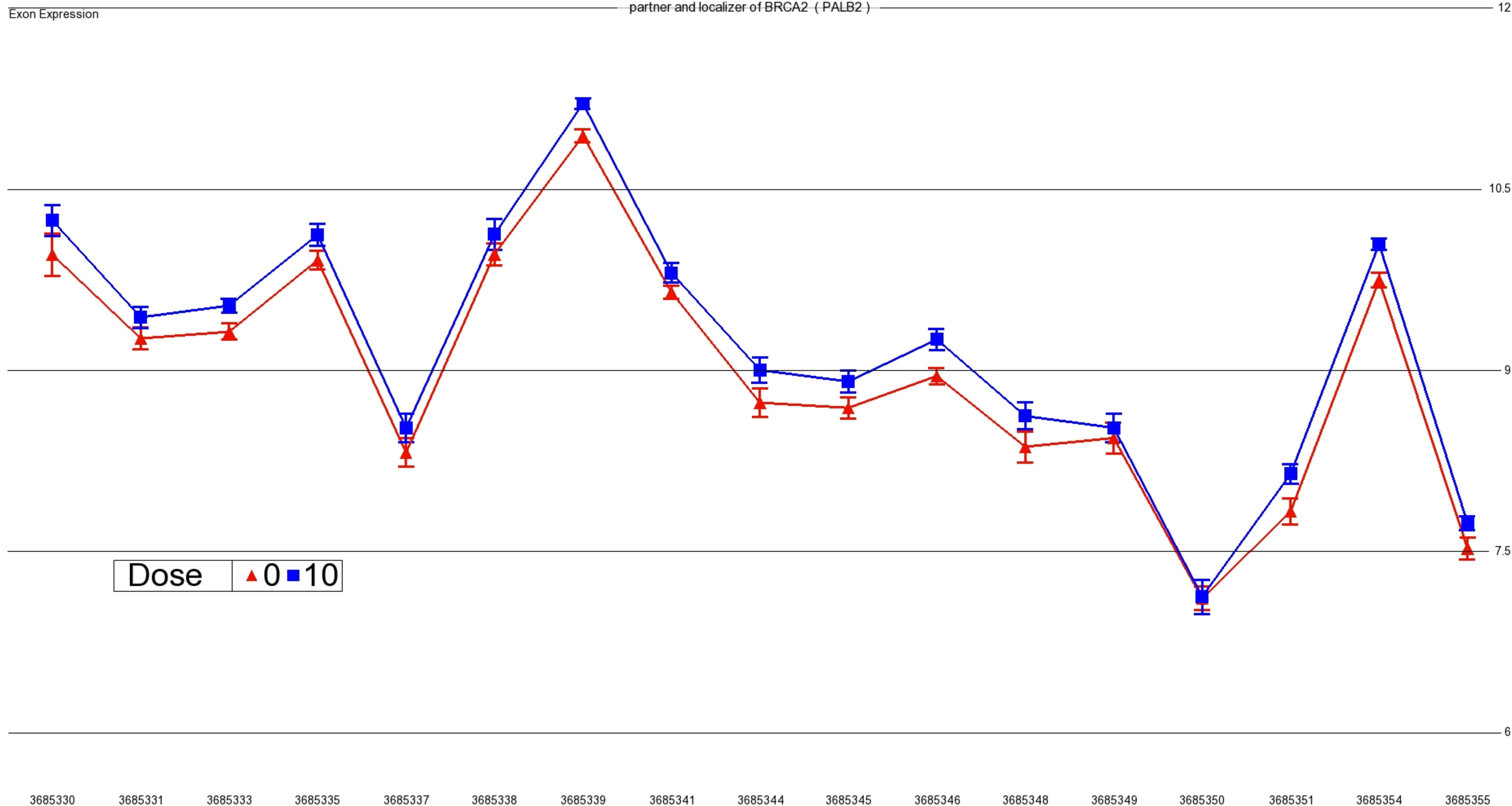

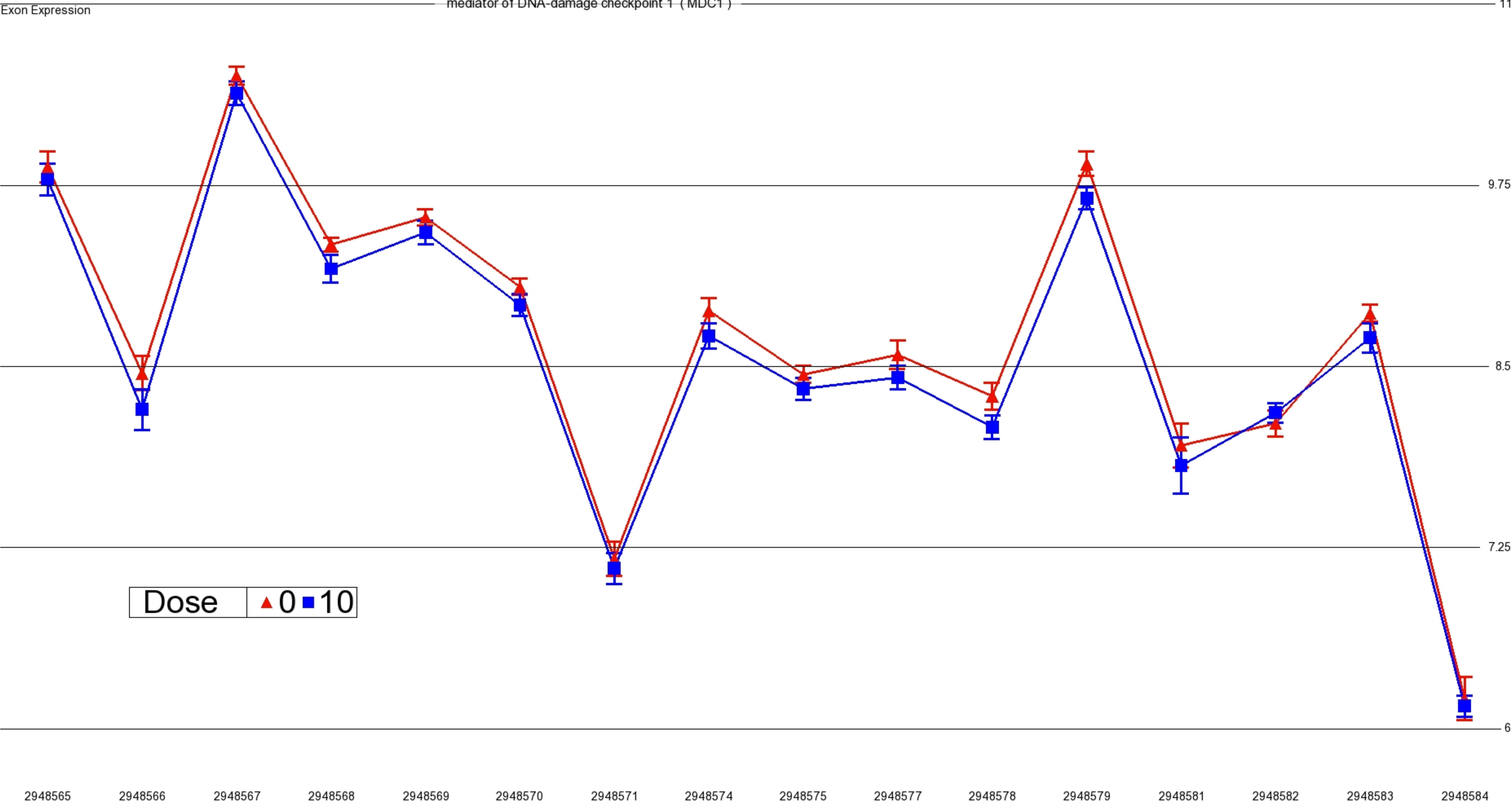

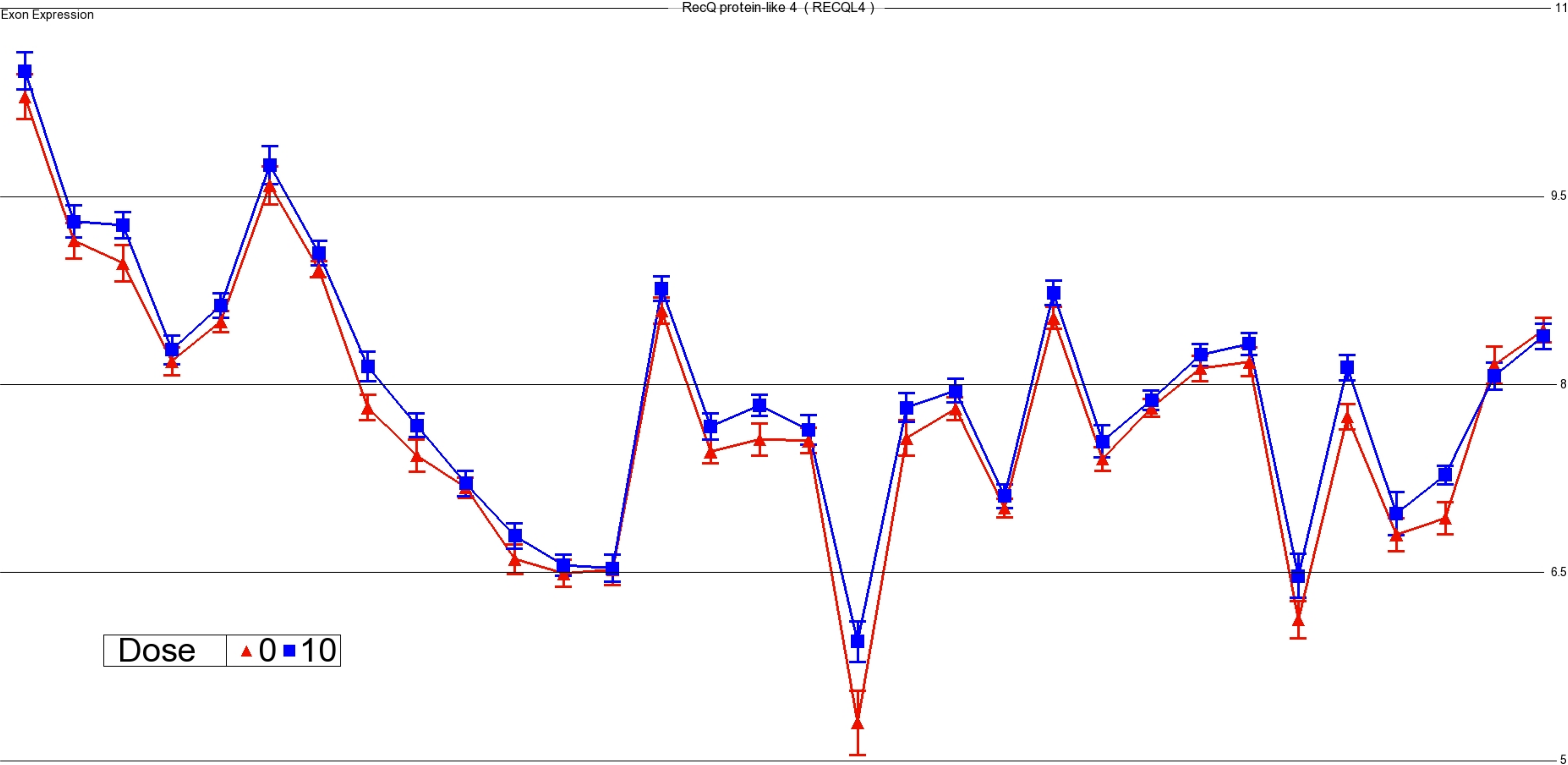

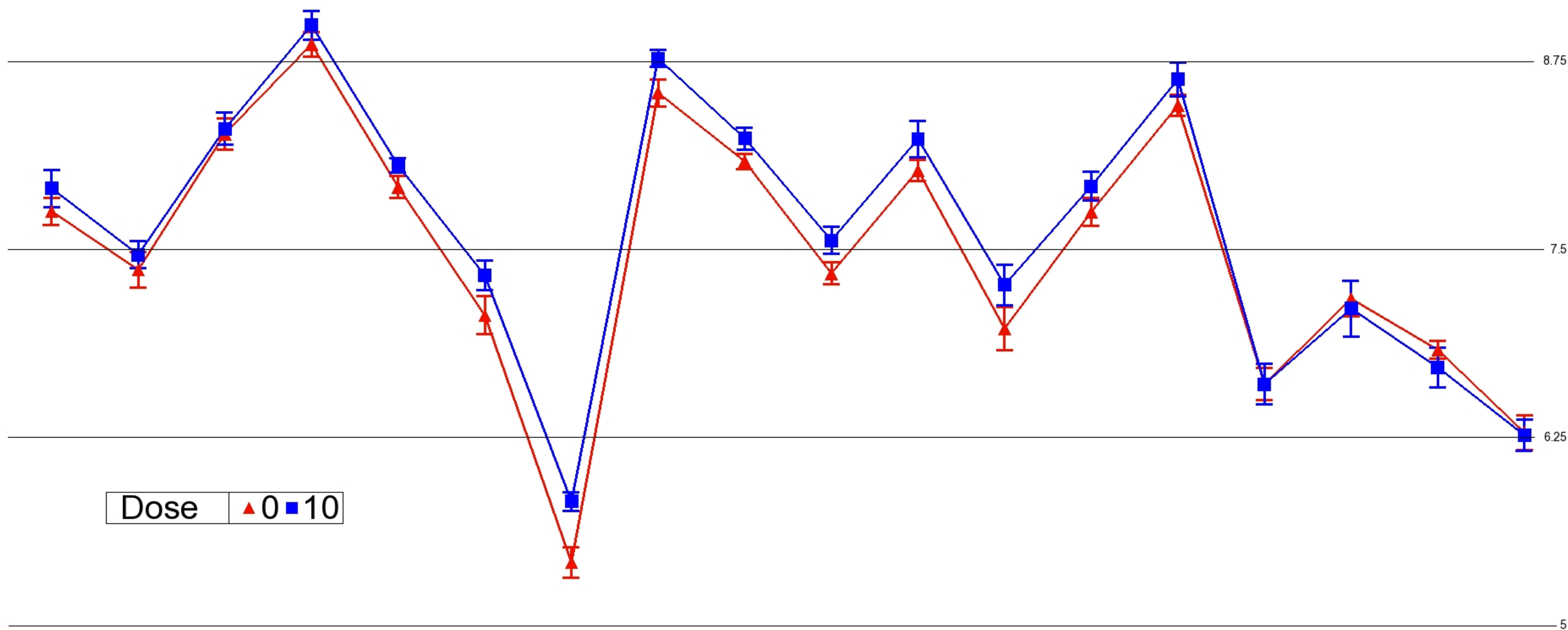

Dose

▲ 0 ■ 10

Supplement: Figure S1 — Modulation of DNA repair genes at the exon level 4 hours after treatment with 10 Gy IR in lymphoblast cell lines. PSR expression levels are plotted for each of the DNA repair genes with a p-value of <0.05 using Partek Genomics Suite statistical package. Relative fluorescence (y-axis; log base 2) is plotted for each PSR (x-axis). Core PSRs are labelled below the graphs. Samples were either sham irradiated (red) or irradiated (blue) with 10 Gy from a 137Cs source. Error bars = SEM (n = 12). (PDF) [file pone.0053358.s001.pdf]

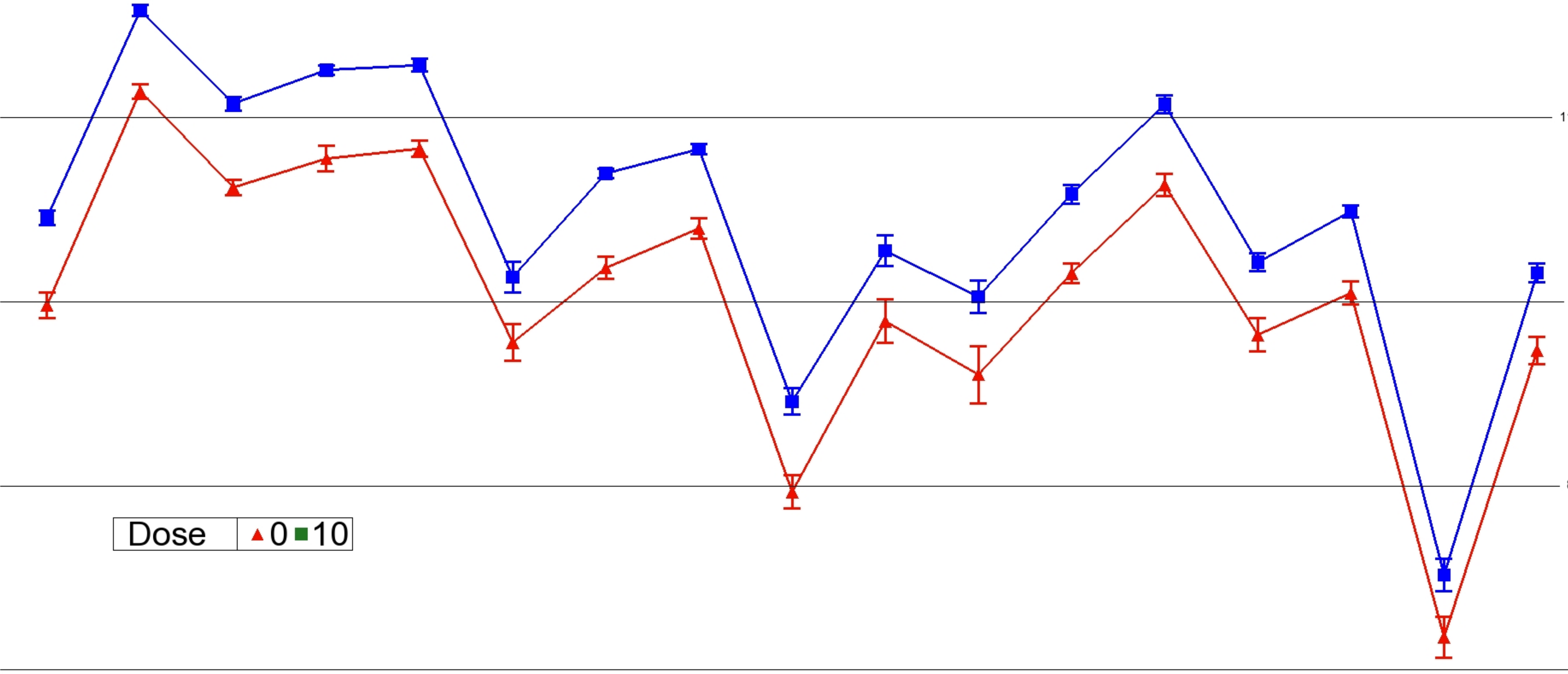

Dose

▲ 0 ■ 10

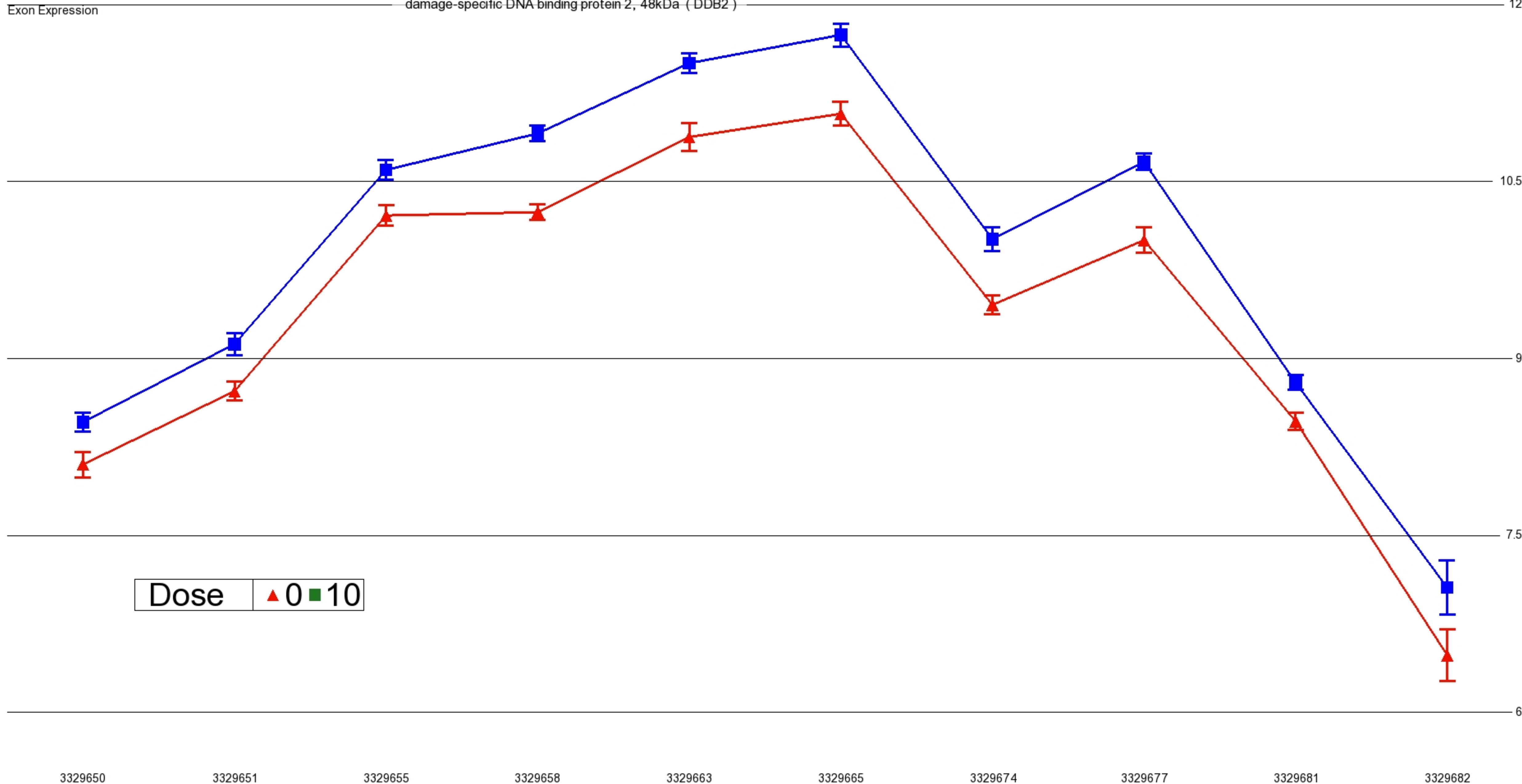

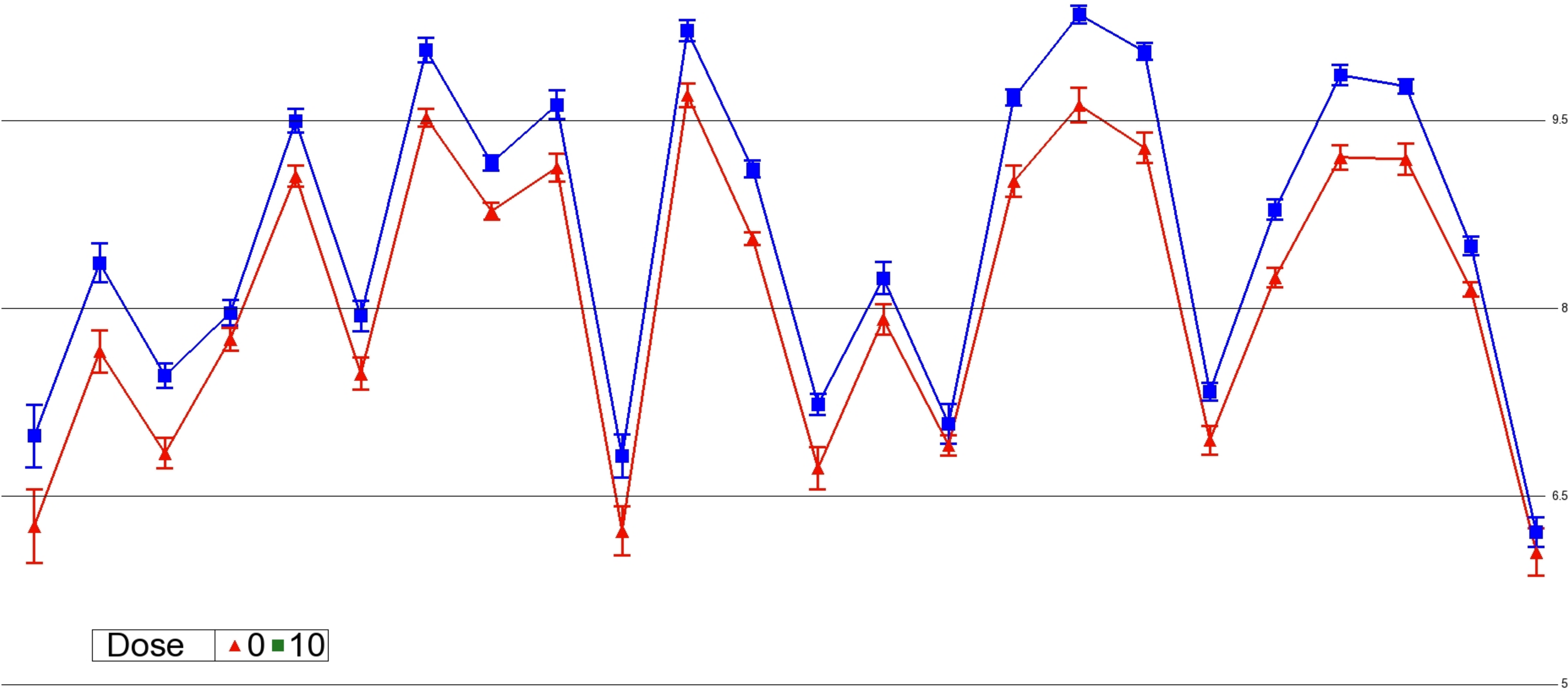

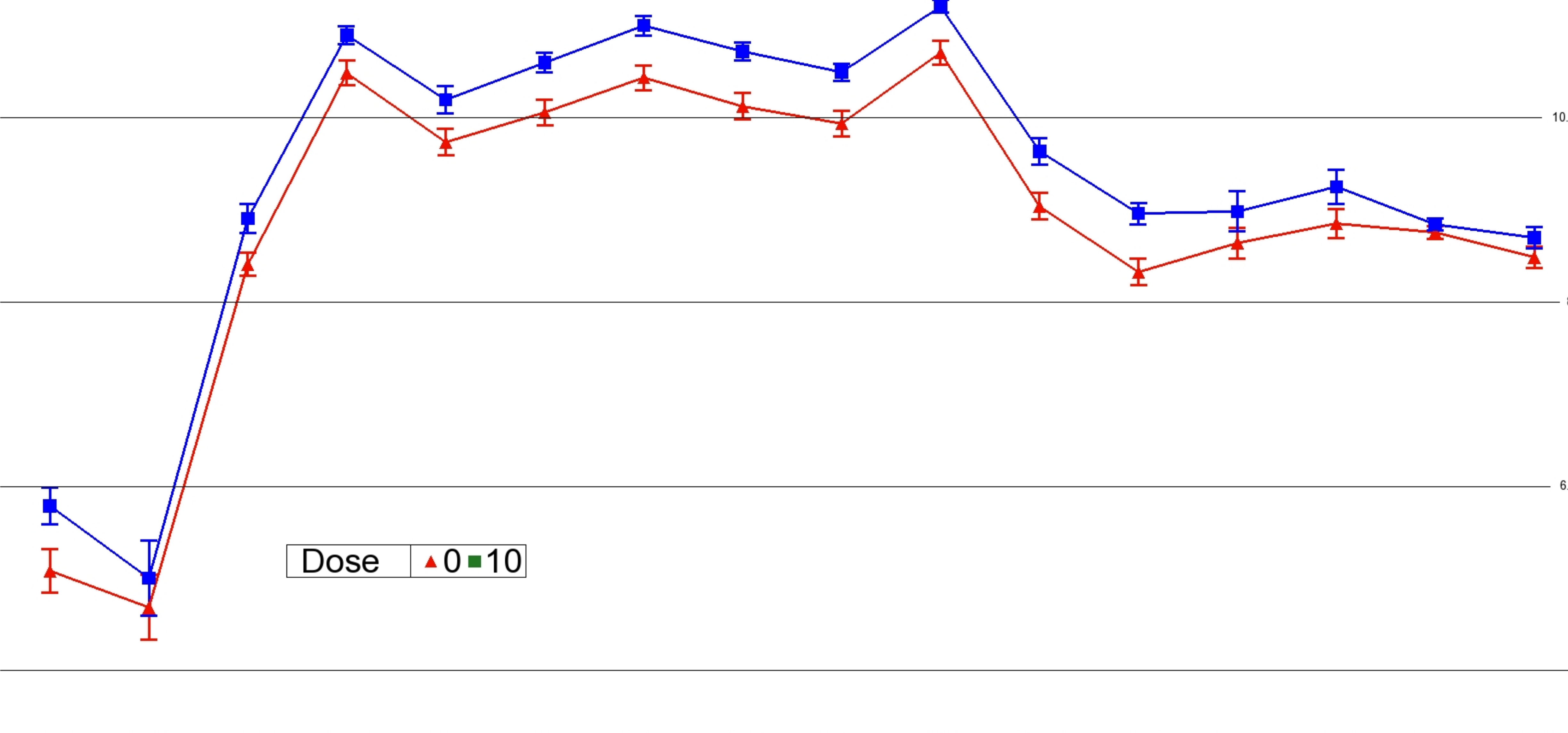

Dose    ▲ 0    ■ 10

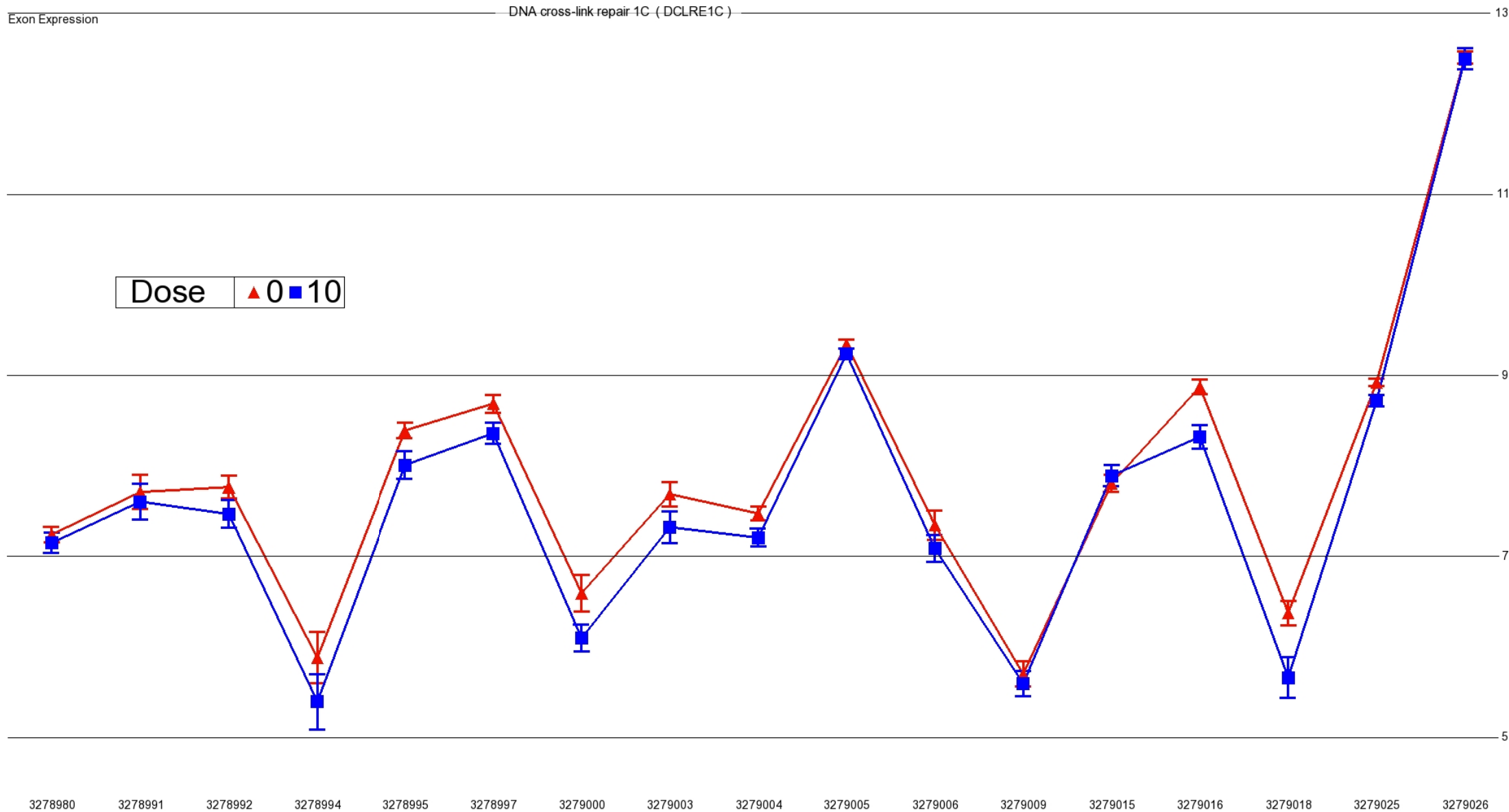

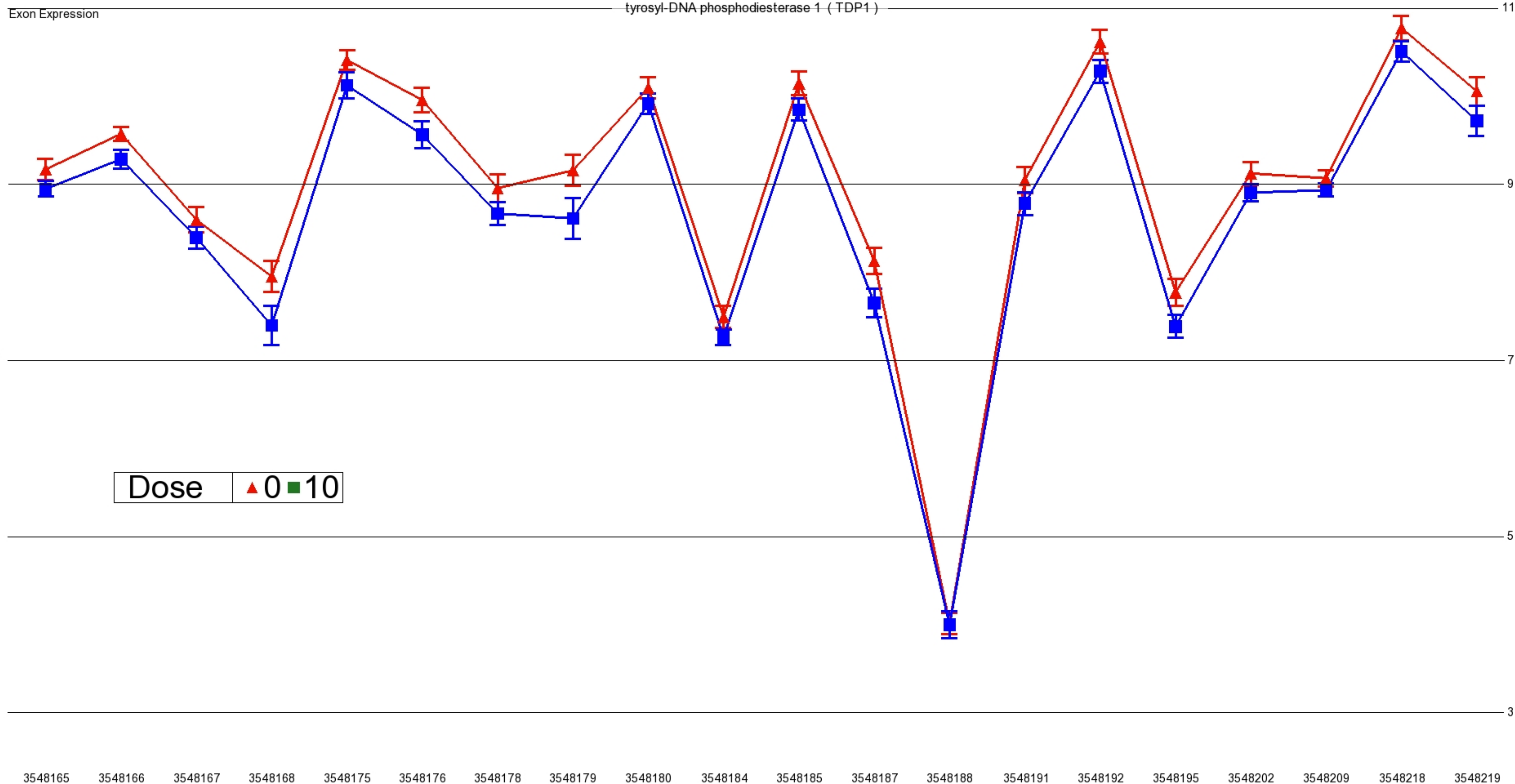

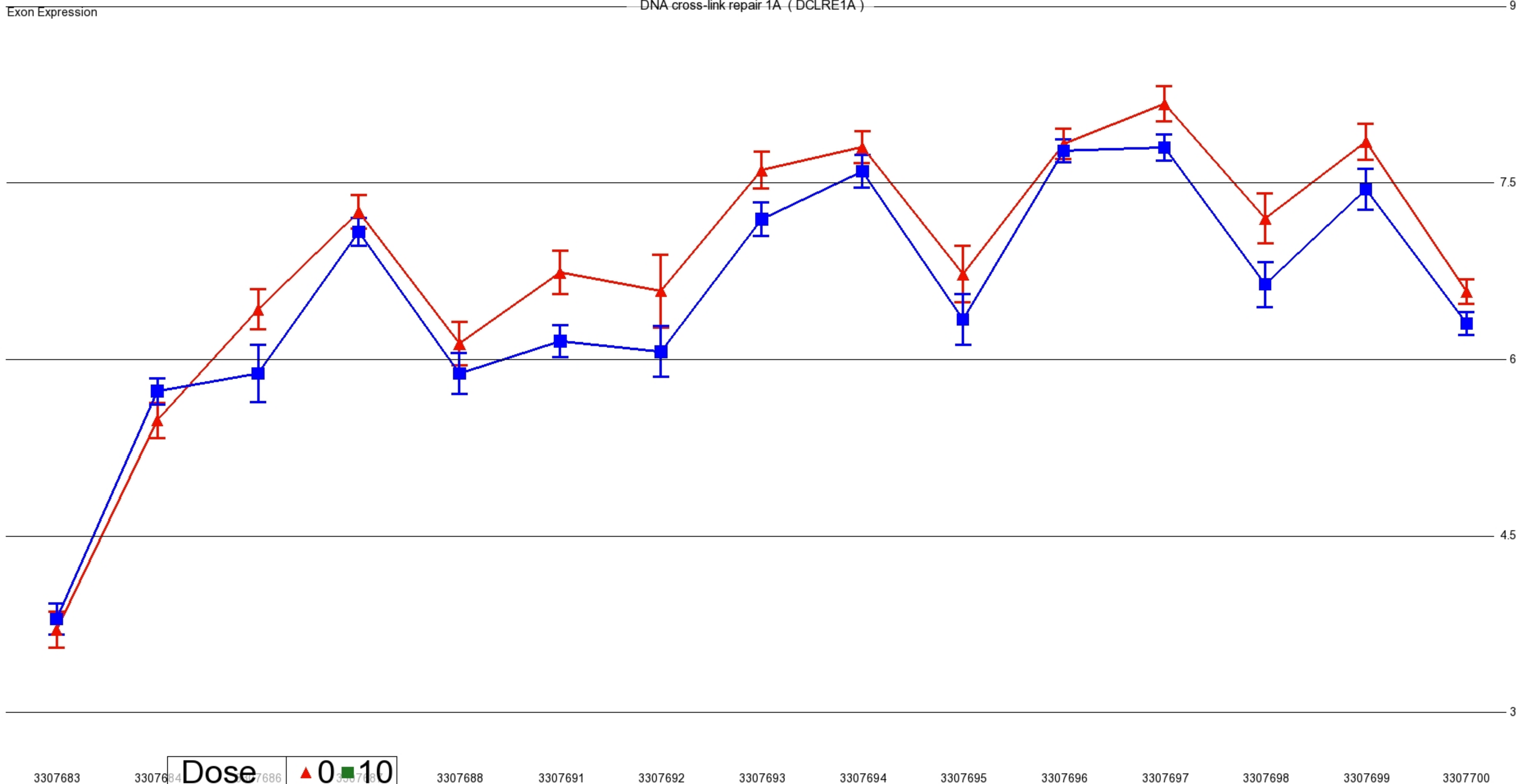

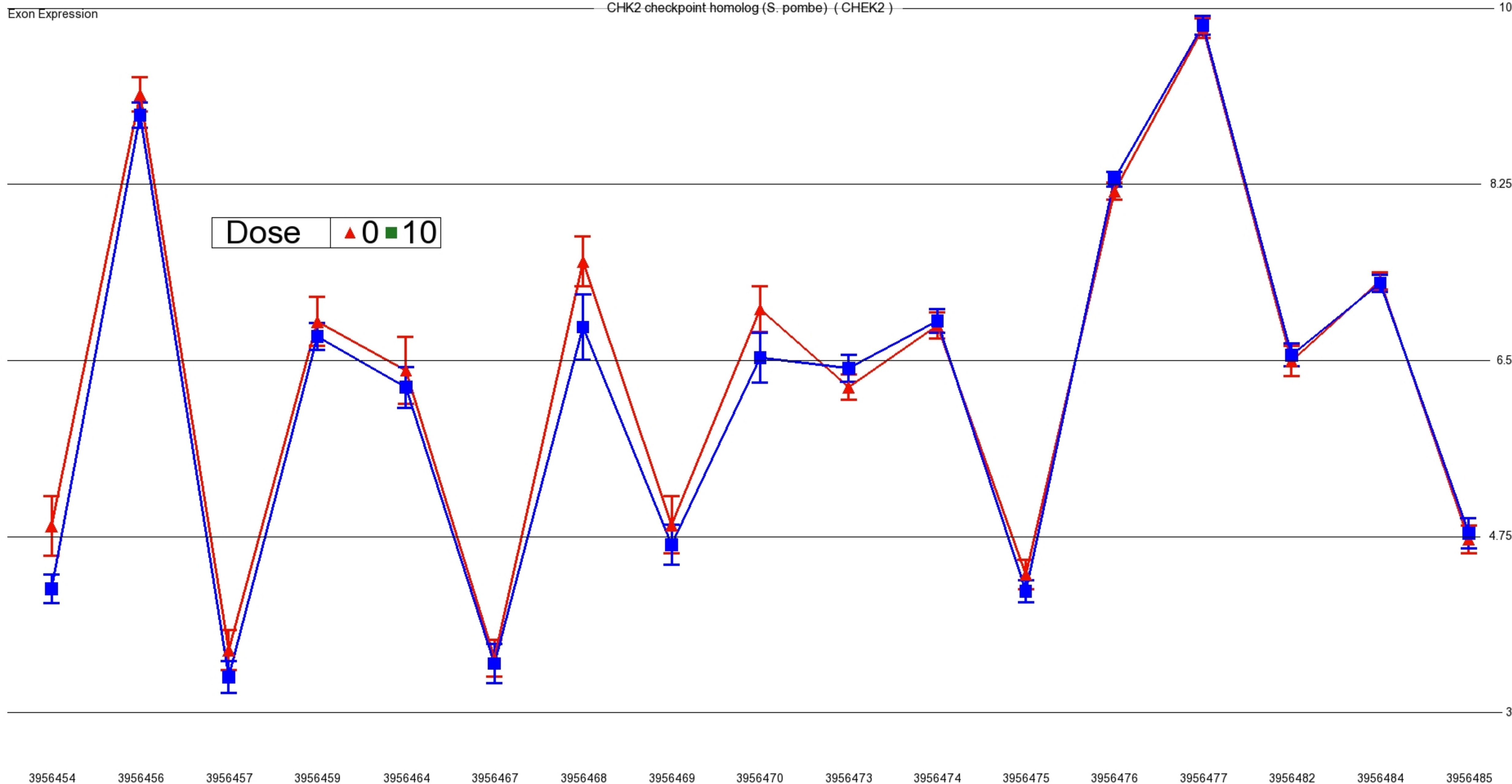

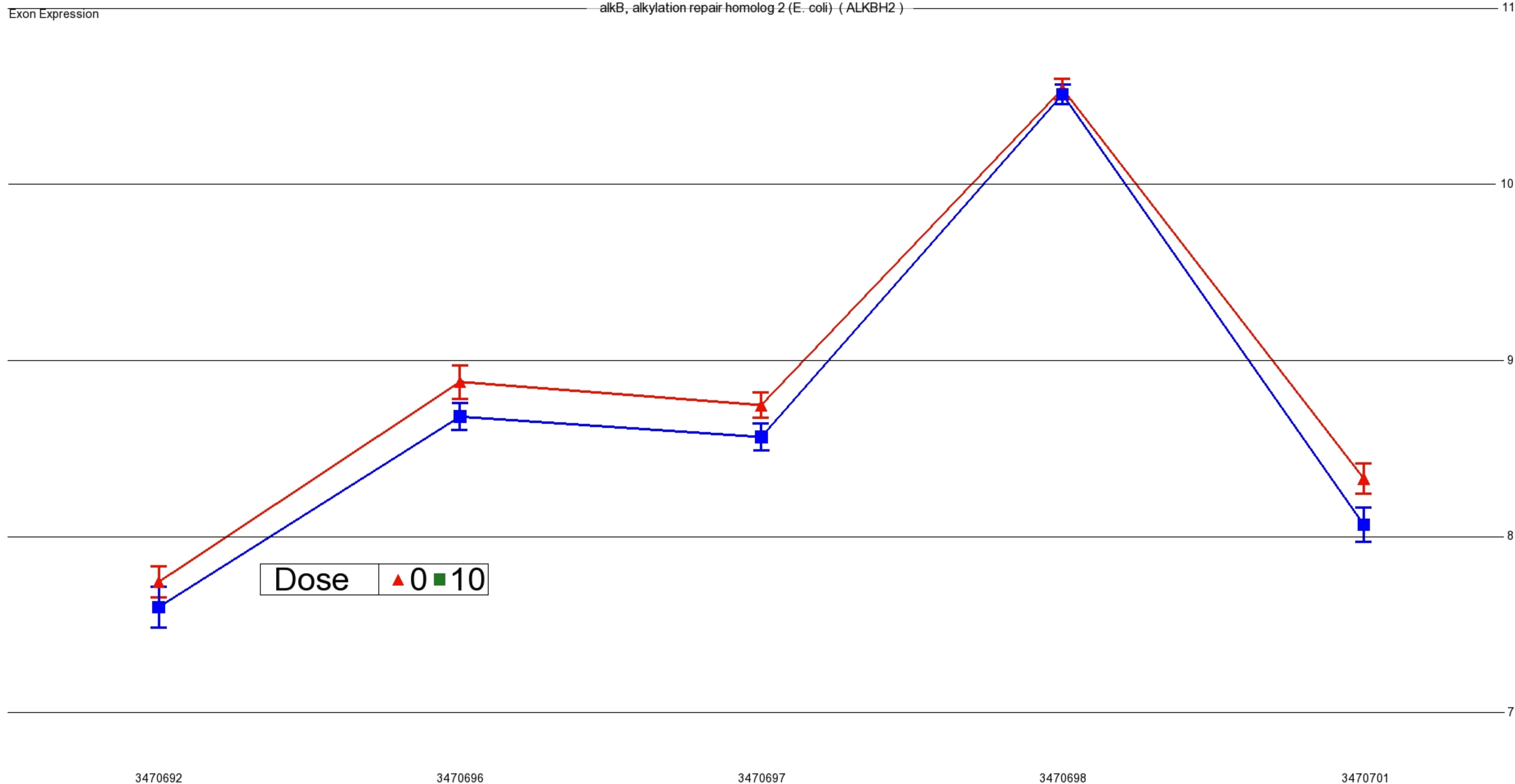

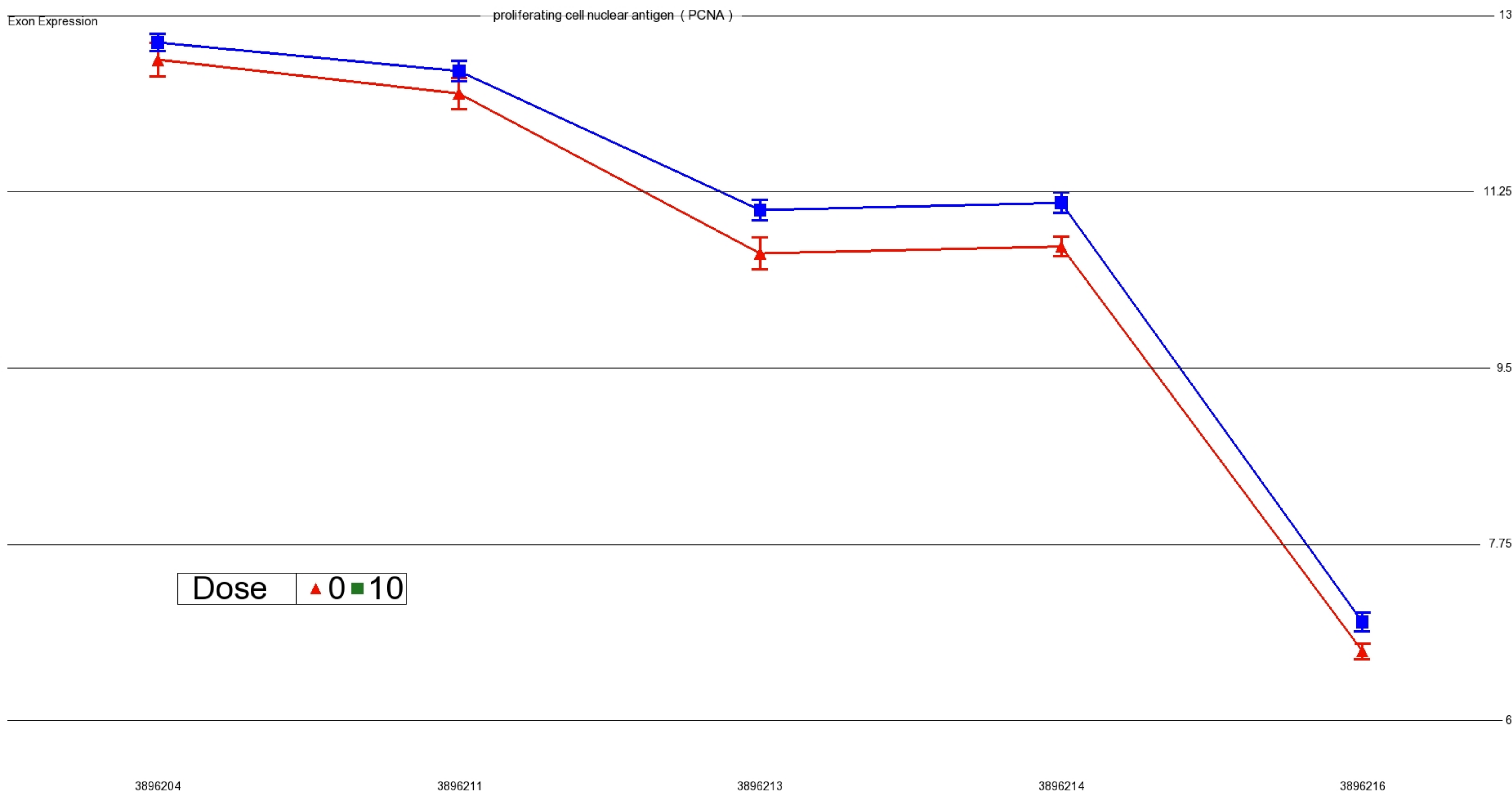

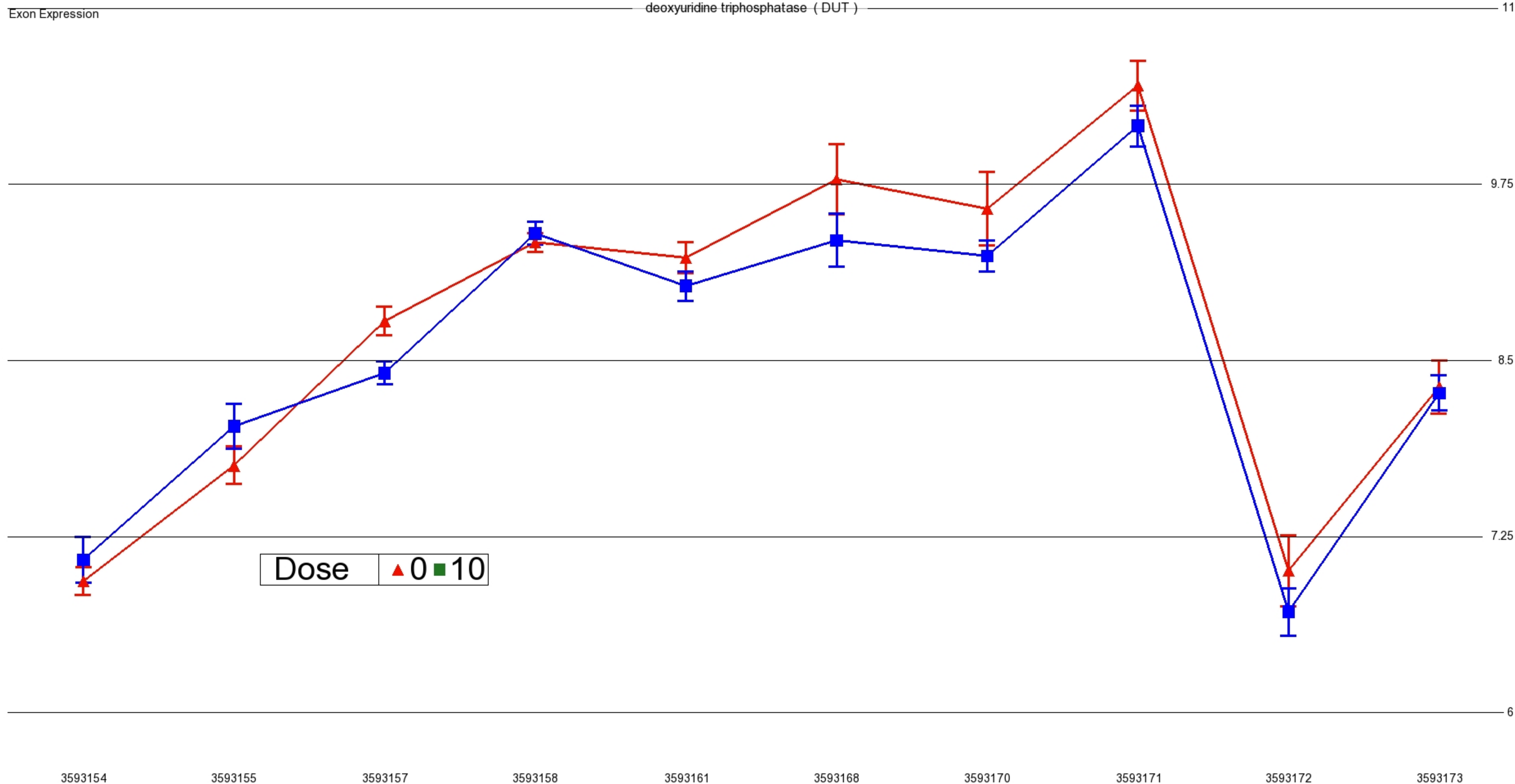

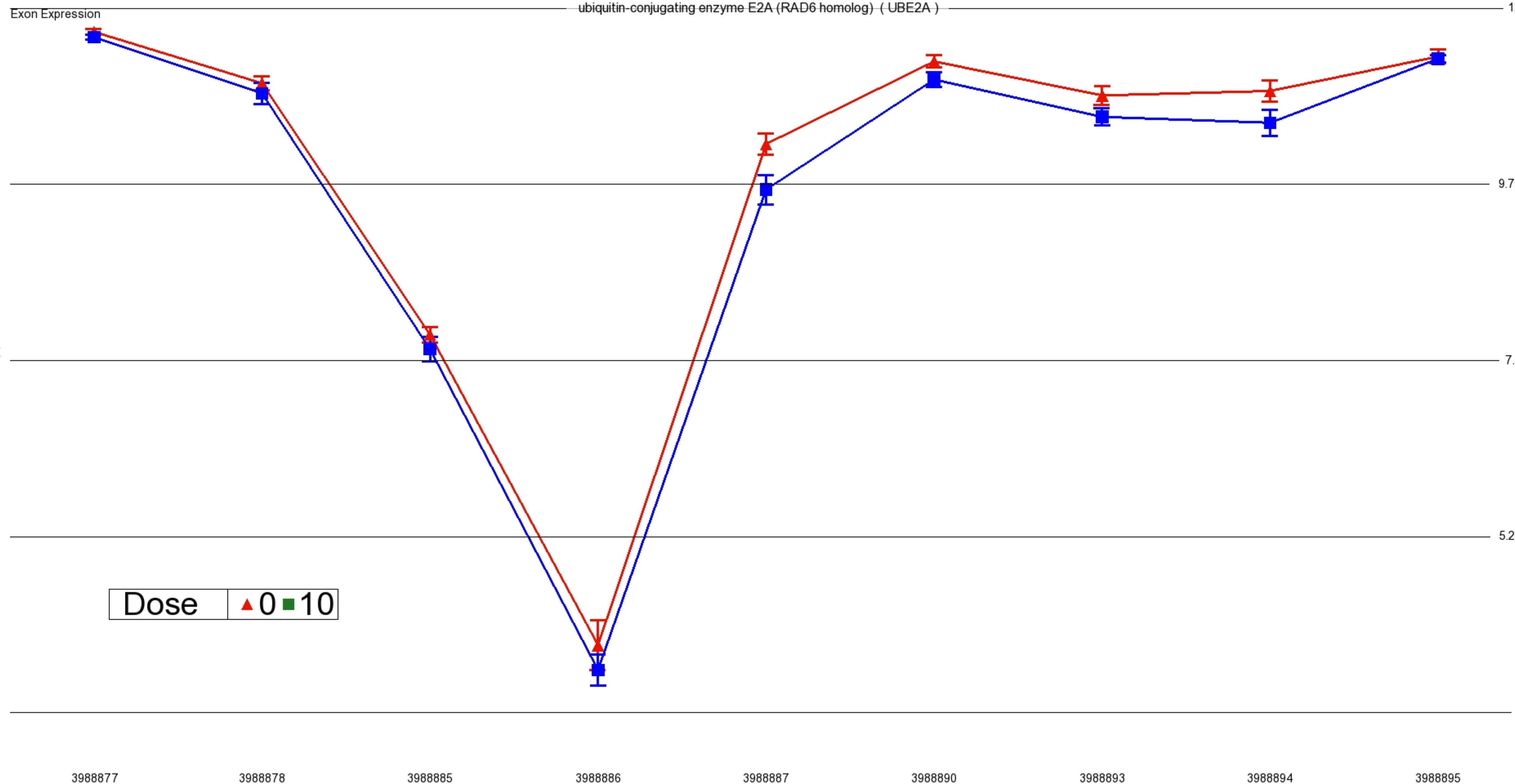

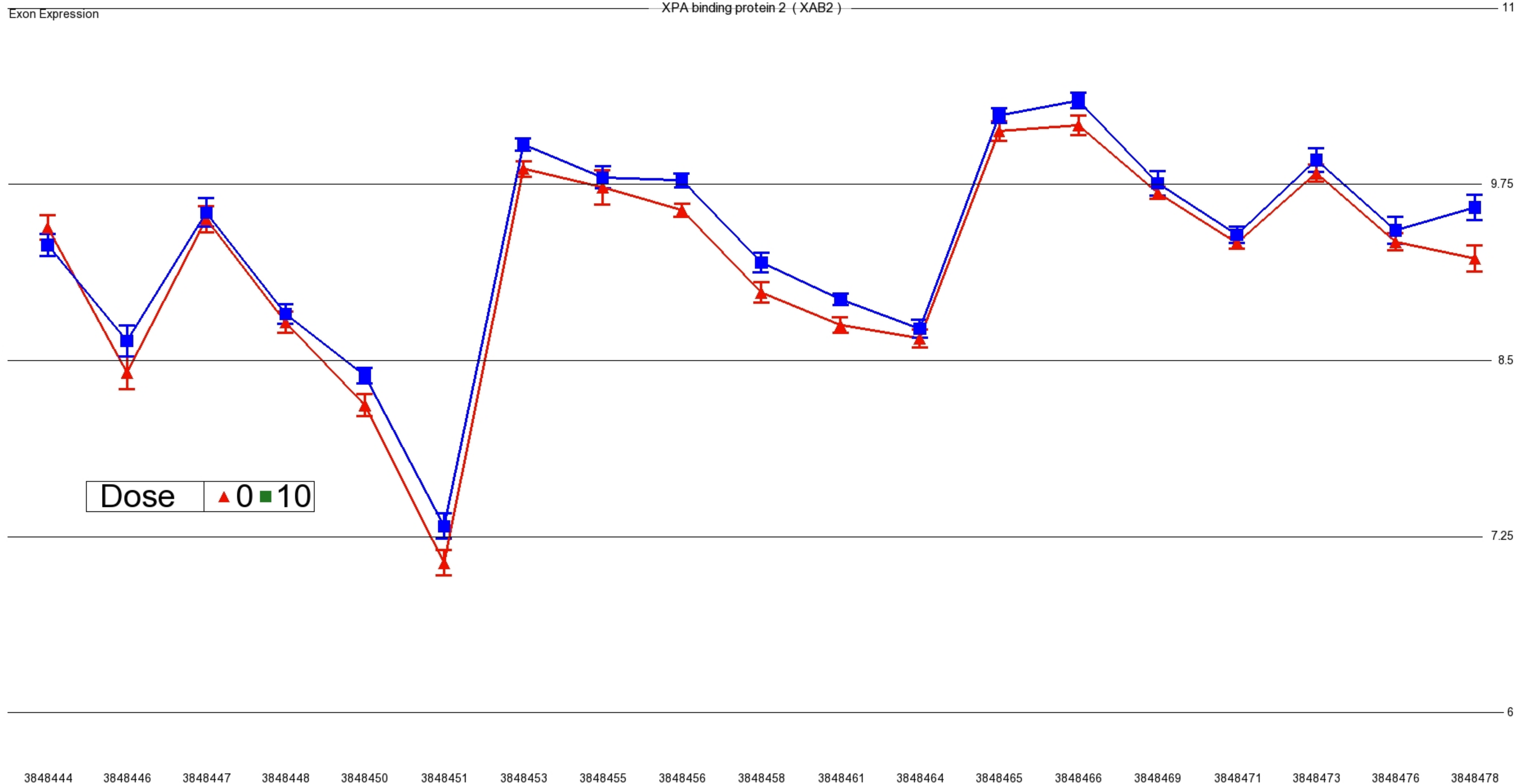

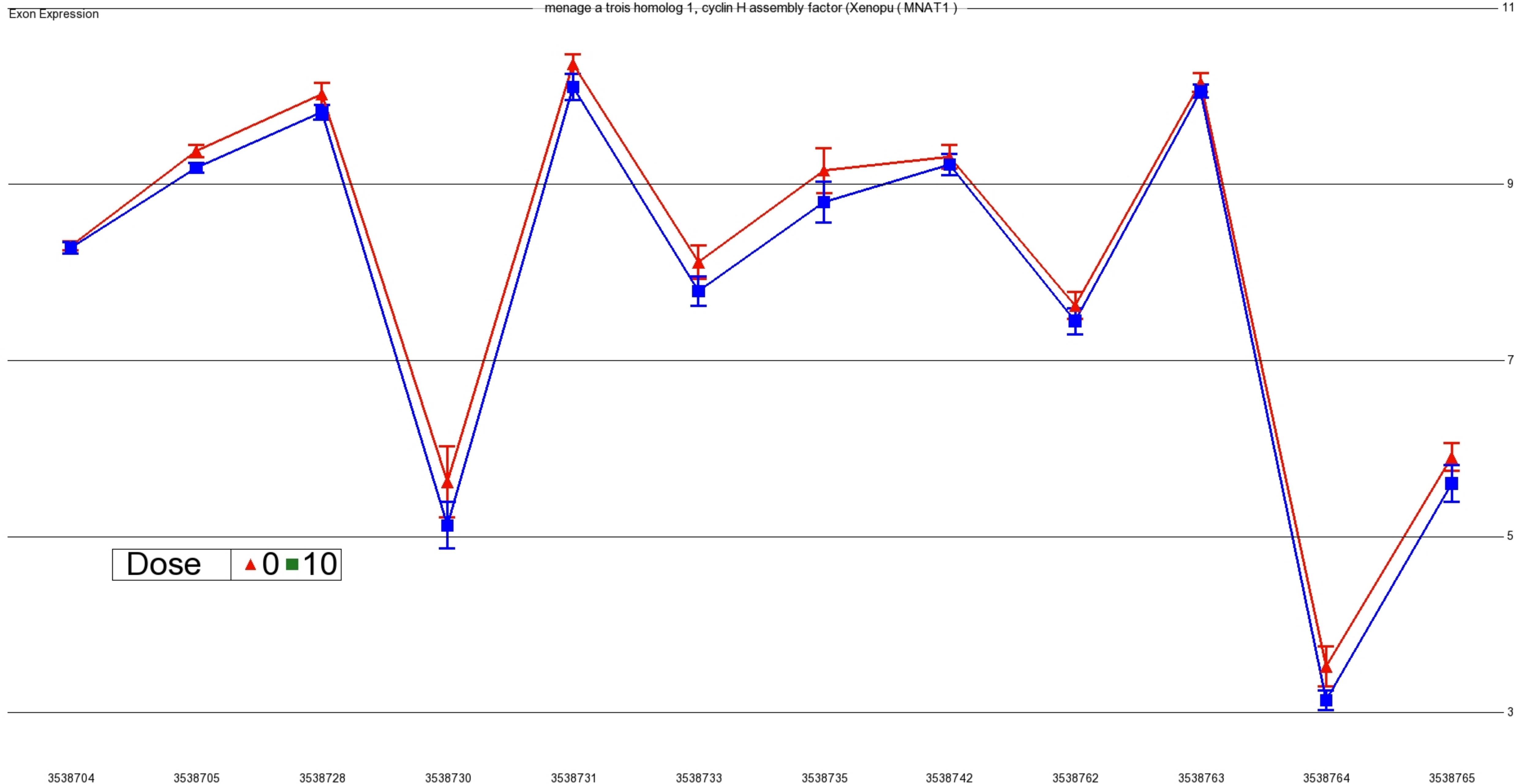

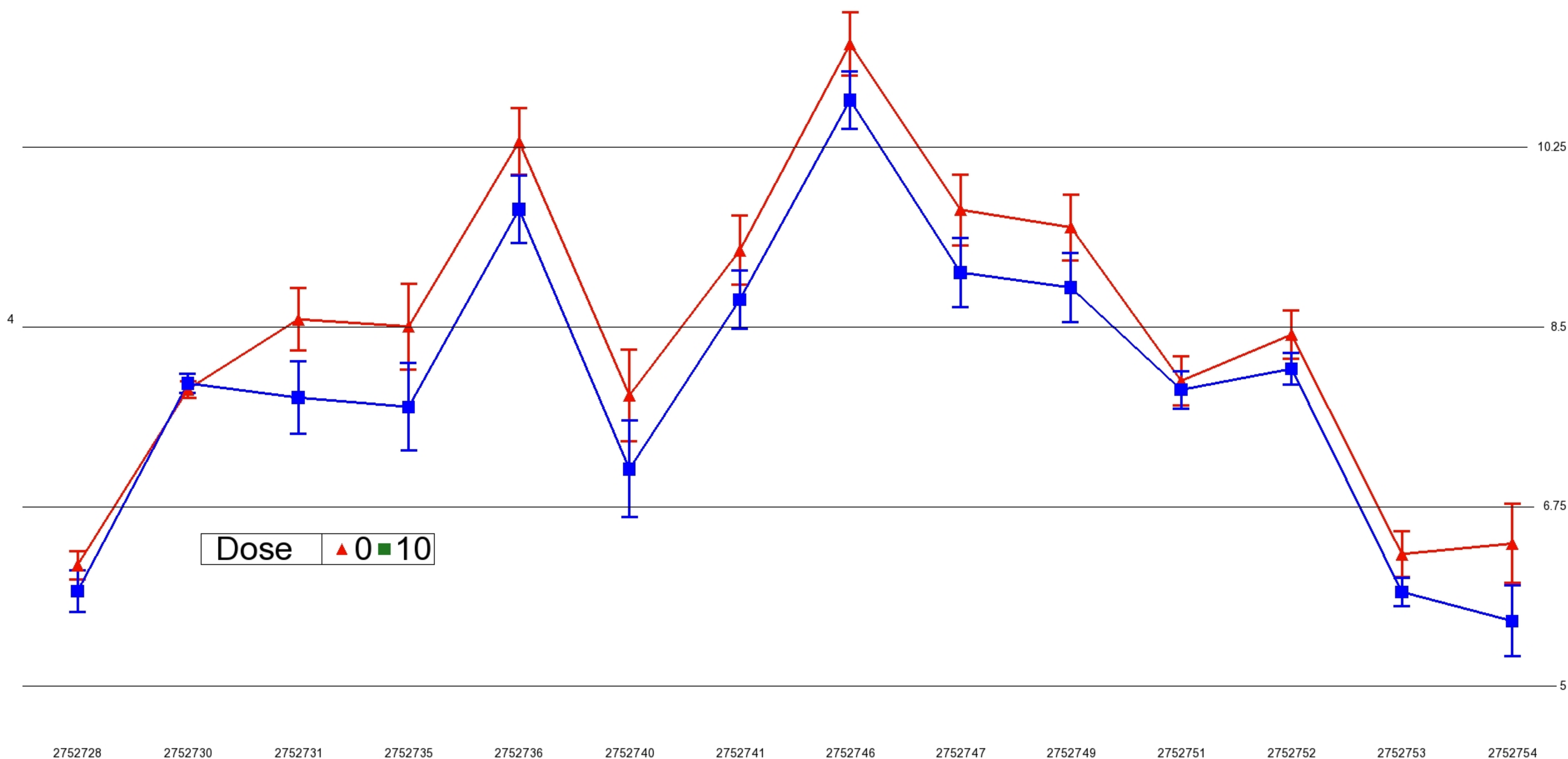

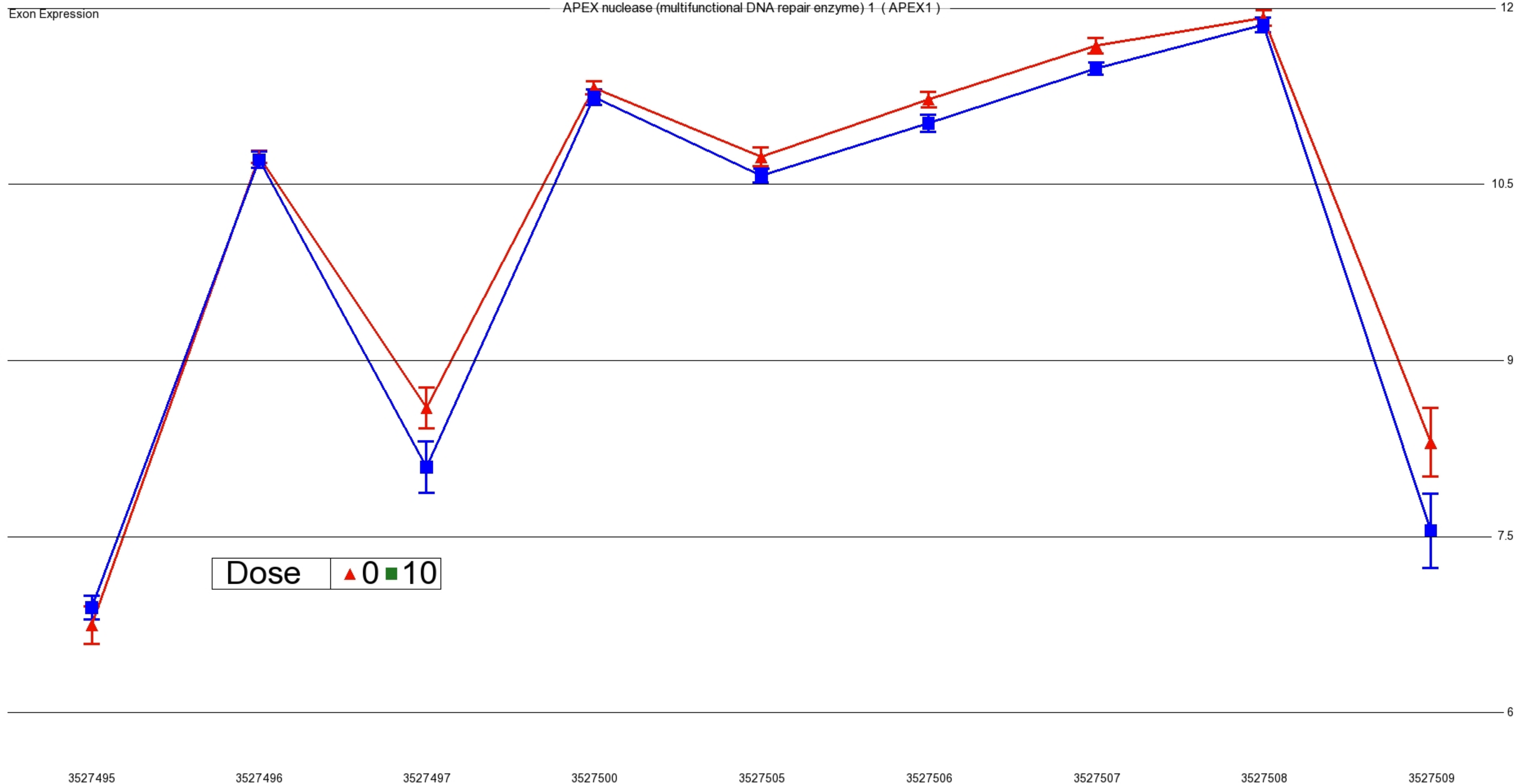

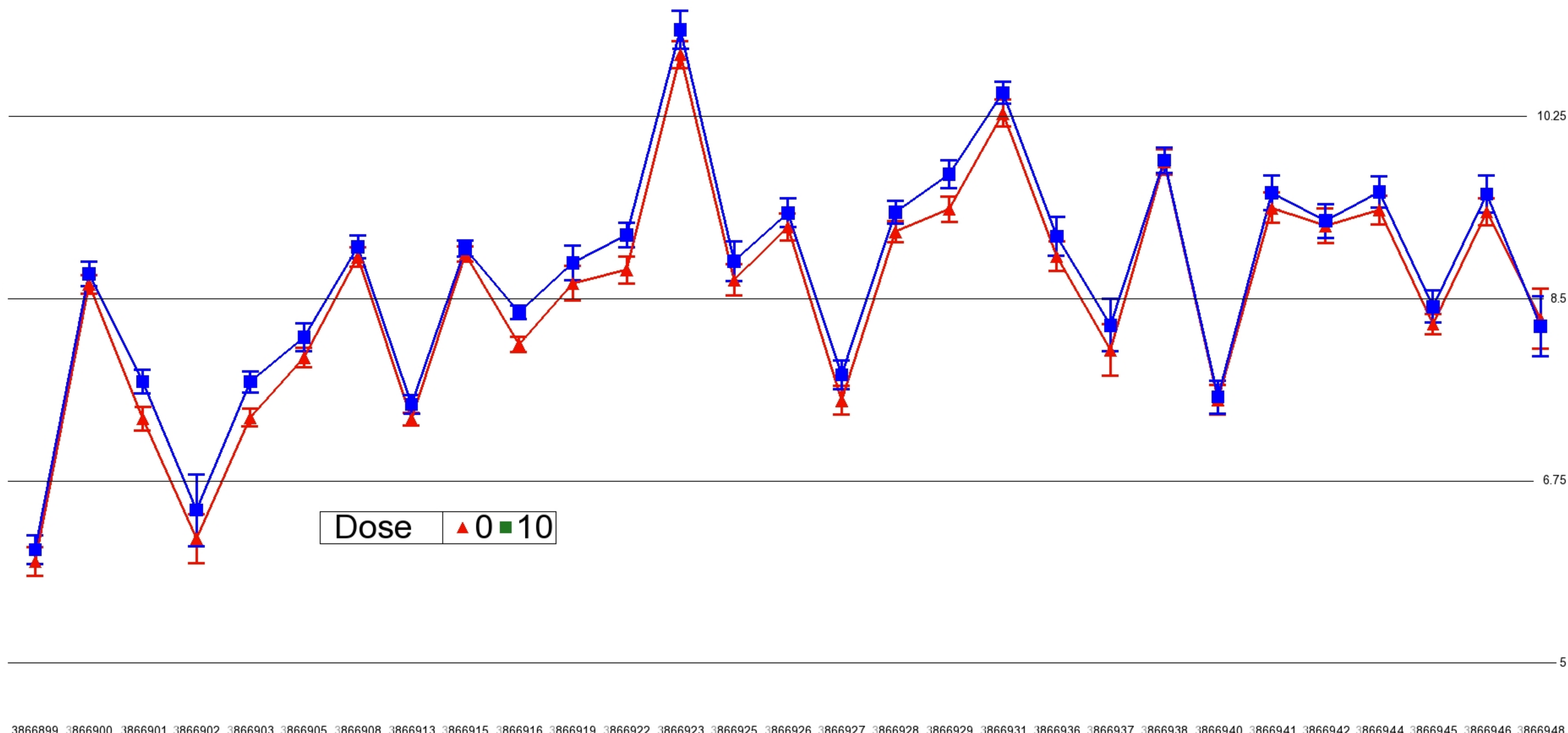

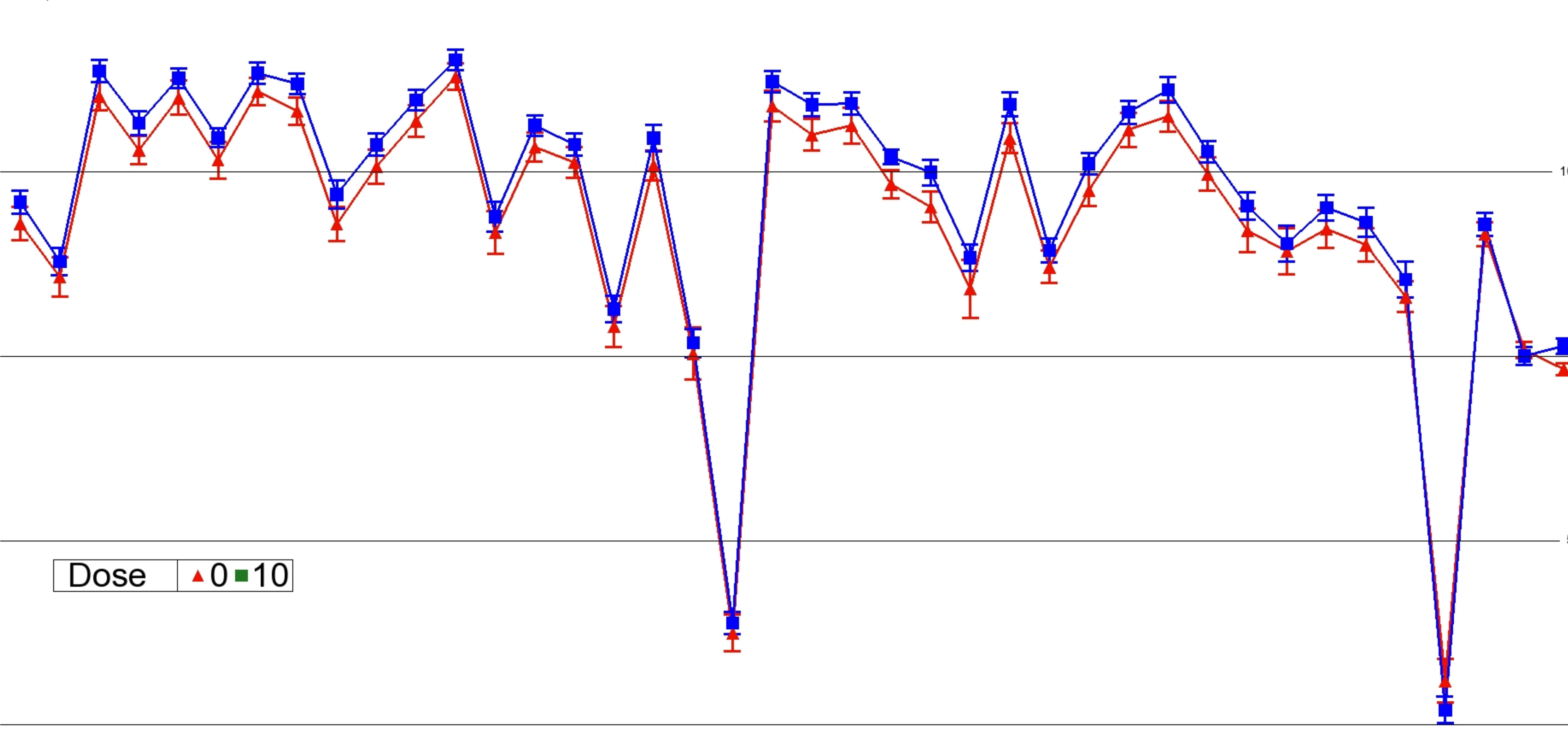

Supplement: Figure S2 — Modulation of DNA repair genes at the exon level 4 hours after treatment with 10 Gy IR in fibroblast cells. PSR expression levels are plotted for each of the DNA repair genes with a p-value of <0.05 using Partek Genomics Suite statistical package. Relative fluorescence (y-axis; log base 2) is plotted for each PSR (x-axis). Core PSRs are labelled below the graphs. Samples were either sham irradiated (red) or irradiated (blue) with 10 Gy from a 137Cs source. Error bars = SEM (n = 12). (PDF) [file pone.0053358.s002.pdf]
