# Supplementary material for: DNA Repair Genes: Alternative Transcription and Gene Expression at the Exon Level in Response to the DNA Damaging Agent, Ionizing Radiation
Source: PLoS One. 2012 Dec 28;7(12):e53358. doi: 10.1371/journal.pone.0053358 (PMC3532210; doi:10.1371/journal.pone.0053358)
Supplement: Figure S3 — Real-time PCR comparison of DNA repair gene expression between LCLs and fibroblasts. Initial levels of transcripts are significantly higher in fibroblasts (FB) for XPC and RRM2B relative to LCLs, but after IR induction, gene expression levels are not significantly different. Values have been normalized to PGK gene expression. The p-values for differences of initial levels between LCL and fibroblast samples are as follows: XPC: p = 0.0025 and for RRM2B: p = 0.0030. (PPTX) [file pone.0053358.s003.pptx]

## Slide 1
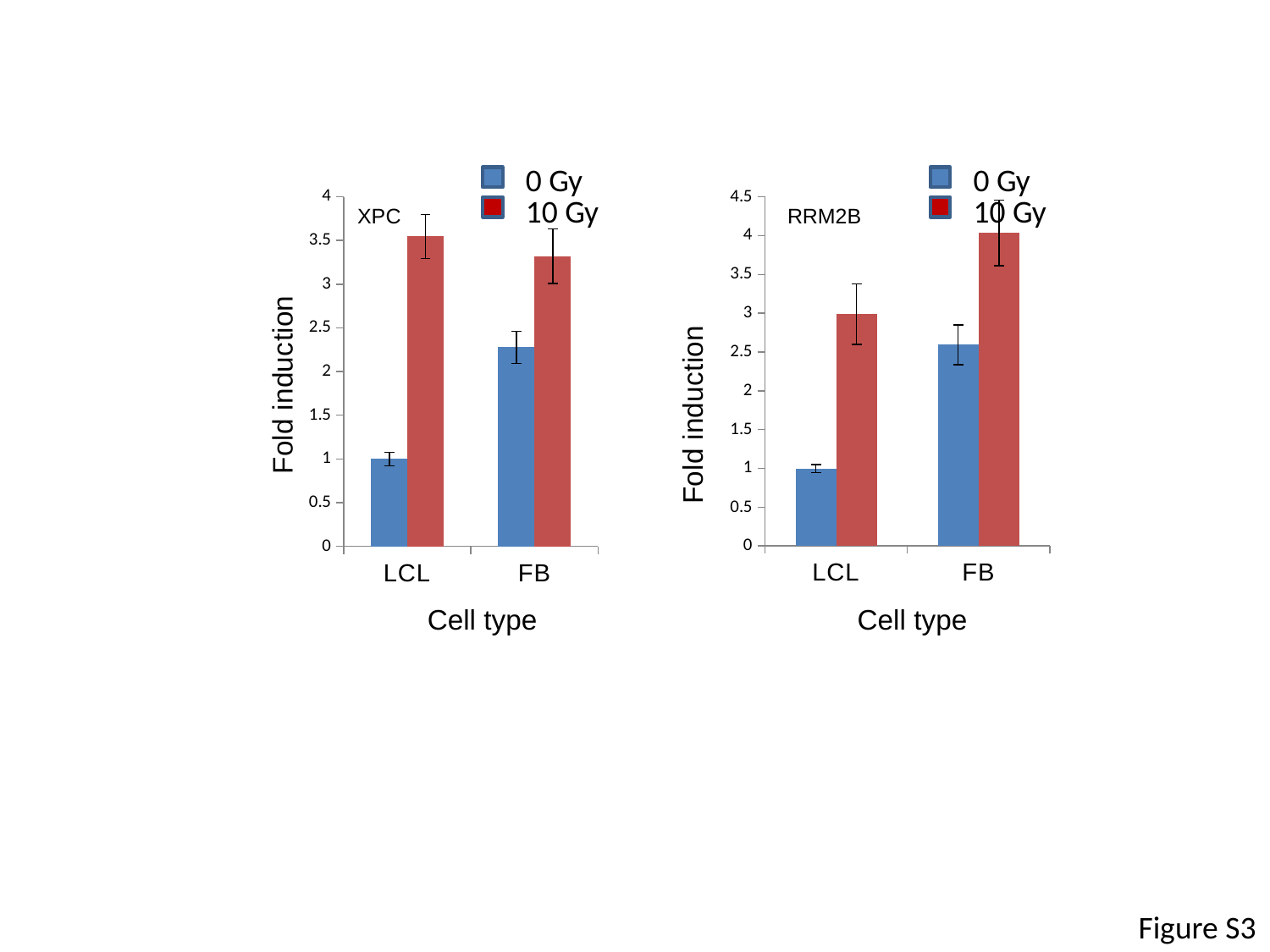

0 Gy
10 Gy
0 Gy
10 Gy
### Chart
| Category | | LCL FB |
|---|---|---|
| LCL | 1.0 | 3.5450590289831623 |
| FB | 2.2769143204107305 | 3.3209908648246134 |
### Chart
| Category | | LCL FB |
|---|---|---|
| LCL | 1.0 | 2.9868618242133897 |
| FB | 2.592980281711754 | 4.035963681808278 |XPC
RRM2B
Cell type
Cell type
Figure S3
