# Supplementary material for: DNA Repair Genes: Alternative Transcription and Gene Expression at the Exon Level in Response to the DNA Damaging Agent, Ionizing Radiation
Source: PLoS One. 2012 Dec 28;7(12):e53358. doi: 10.1371/journal.pone.0053358 (PMC3532210; doi:10.1371/journal.pone.0053358)
Supplement: Figure S4 — Sequence analysis of the 5′RACE amplicons for RRM2B (A) and XPC (B). Sequences homologous to the primers used for their amplifcation are indicated by long horizontal arrows below the sequence. The adaptor sequences are shown in capital letters. The vertical arrows indicate the start site for the capped mRNA transcript. This is nucleotide 35 of NCBI RRM2B sequence NM_001172477 (A), and at nucleotide 261 of NCBI XPC sequence NM_004628 (B). (PPTX) [file pone.0053358.s004.pptx]

## Slide 1
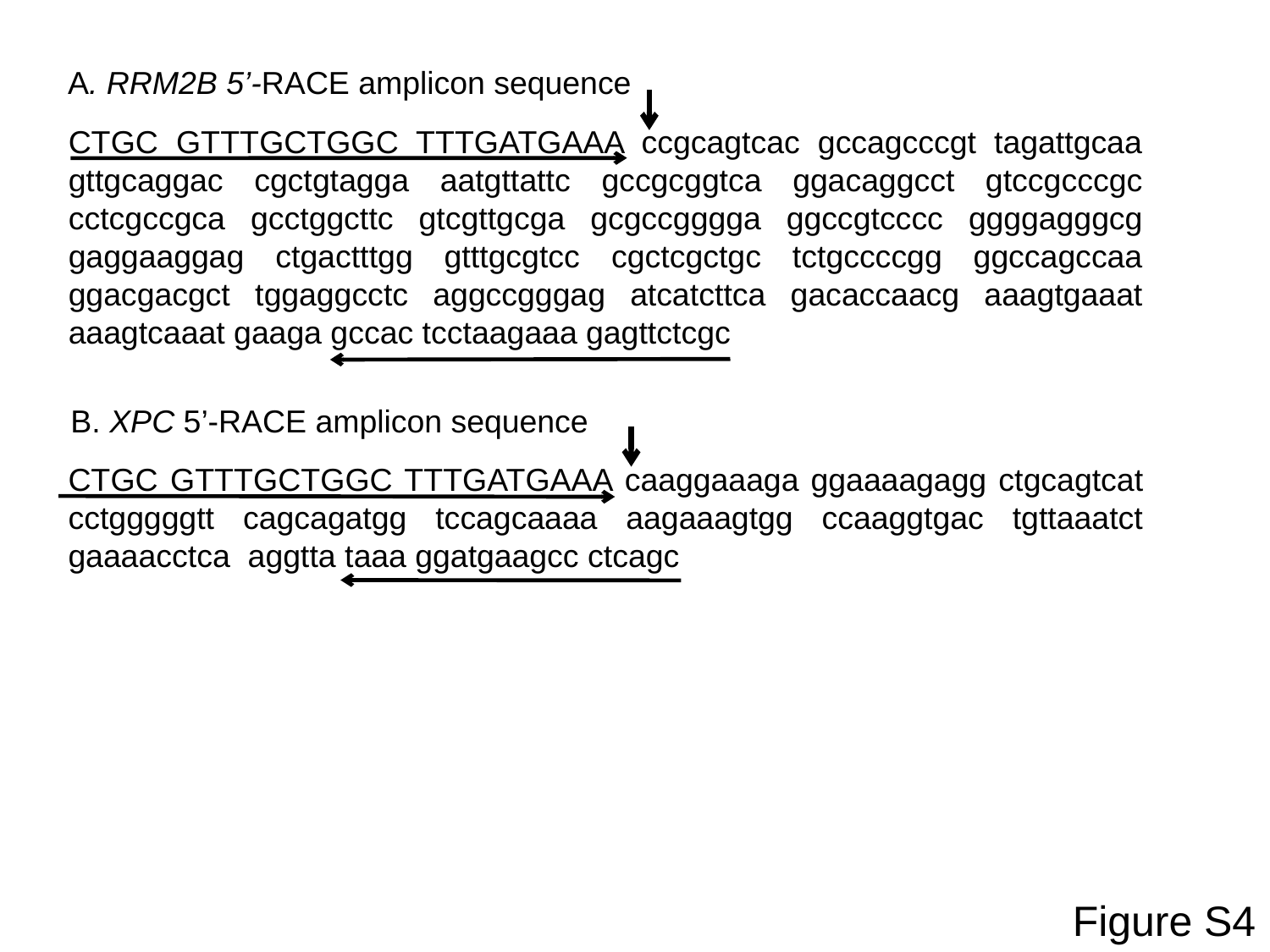

A. RRM2B 5’-RACE amplicon sequence
# CTGC GTTTGCTGGC TTTGATGAAA ccgcagtcac gccagcccgt tagattgcaa gttgcaggac cgctgtagga aatgttattc gccgcggtca ggacaggcct gtccgcccgc cctcgccgca gcctggcttc gtcgttgcga gcgccgggga ggccgtcccc ggggagggcg gaggaaggag ctgactttgg gtttgcgtcc cgctcgctgc tctgccccgg ggccagccaa ggacgacgct tggaggcctc aggccgggag atcatcttca gacaccaacg aaagtgaaat aaagtcaaat gaaga gccac tcctaagaaa gagttctcgc
CTGC GTTTGCTGGC TTTGATGAAA caaggaaaga ggaaaagagg ctgcagtcat cctgggggtt cagcagatgg tccagcaaaa aagaaagtgg ccaaggtgac tgttaaatct gaaaacctca aggtta taaa ggatgaagcc ctcagc
B. XPC 5’-RACE amplicon sequence
Figure S4
